# Pseudosymmetry and high *Z*′ structures: the case of *rac*-(2*R*,2′*R*,5′*S*)-2-methyl-5′-[(1*R*,2*R*,5*S*,5′*S*)-1,4,4,5′-tetra­methyl­dihydro-3′*H*-3,8-dioxa­spiro­[bi­cyclo­[3.2.1]octane-2,2′-furan]-5′-yl]-3,4,1′,2′,3′,4′-hexa­hydro-[2,2′-bi­furan]-5(2*H*)-one

**DOI:** 10.1107/S2056989017010805

**Published:** 2017-08-01

**Authors:** Vincenzo Piccialli, Angela Tuzi, Roberto Centore

**Affiliations:** a Dipartimento di Scienze Chimiche, Università degli Studi di Napoli ’Federico II’, Complesso di Monte S. Angelo, Via Cinthia, 80126 Napoli, Italy

**Keywords:** crystal structure, pseudosymmetry, high *Z*′ compounds, poly-THF compounds, spiro compounds

## Abstract

The title compound, C_22_H_34_O_6_, is one of the products obtained by oxidation of squalene with the catalytic system RuO_4_(cat.)/NaIO_4_. It crystallizes in the *P*-1 space group, with four crystallographically independent mol­ecules related by a pseudo-*C*_2_ symmetry axis. The structural analysis also shows that the title compound is isomeric with two products previously reported in the literature and that are obtained by the same reaction procedure. In particular, out of the seven chiral C atoms present in the mol­ecule, the title compound shows the opposite configuration at, respectively, four and two chiral centres with respect to the isomeric compounds.

## Abstract 2

The title compound crystallizes in the *P* 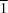 space group, with four crystallographically independent mol­ecules.

## Chemical context   

The search for new lead compounds is a major goal in drug discovery and chemistry of materials (Teta *et al.*, 2013 ▸). Our group has long been involved in the synthesis of new biologically active heterocyclic compounds, including purine nucleoside analogues (D’Errico *et al.*, 2012*a* ▸,*b* ▸; Oliviero *et al.*, 2008 ▸, 2010*a* ▸,*b* ▸), cyclic ethers (Piccialli *et al.*, 2007 ▸, 2013 ▸), triazoles (Iovine *et al.*, 2014 ▸) and spiro­ketal compounds (Piccialli *et al.*, 2009 ▸; Piccialli, 2014 ▸). Heterocyclic compounds as building blocks for advanced materials have also been studied, including fused-ring heteroarenes (Centore *et al.*, 1999 ▸; Carella *et al.*, 2007 ▸) and N-rich aromatics (Centore *et al.*, 2013 ▸). In particular, we have recently reported the synthesis of structurally new spiro­ketal compounds through ruthenium and chromium chemistry (Piccialli *et al.*, 2009 ▸).

**Figure 1:**
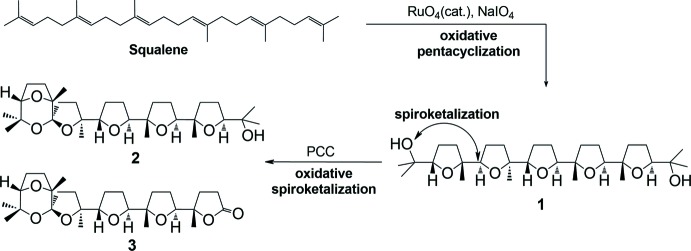
Scheme of synthesis showing the oxidative cyclization of squalene with RuO_4_ and post-cyclization oxidative chemistry.

As a continuation of our efforts in this area, we report here the isolation of the title compound from the oxidation of squalene with the catalytic system RuO_4_/NaIO_4_. In particular, the stereoselective polycyclization of squalene with catalytic amounts of RuO_4_ (Fig. 1 ▸) (Bifulco *et al.*, 2003 ▸) allows penta-THF **1** to be obtained in a straightforward way and high yields (50% for five consecutive cyclization steps; 87% *per* cyclization step) through a unique oxidative cascade process. In this way, multi-gram amounts of this substance can easily be obtained from a cheap starting material. Compound **1** has been used as the starting material for the synthesis of a number of new poly-THF and spiro­ketal substances (**2–8**, Figs. 1 ▸ and 2 ▸), among which compounds **2** and **3** (Fig. 1 ▸) that have shown anti-cancer activity against ovarian (HEY) and breast cancer-derived (BT474) cell lines (Piccialli *et al.*, 2009 ▸).

The title compound is a stereoisomer of two spiro­ketal compounds previously reported by us (Piccialli *et al.*, 2009 ▸, 2017 ▸). The determination of the configuration of the numerous stereogenic centres belonging to polycyclic polyether compounds such as the title compound, which contains seven chiral carbons, can be a challenging task. Although NMR data generally provide pivotal information on the stereostructure of such substances, definitive confirmation has very often required total synthesis or X-ray diffraction analysis, as experienced by us and reported by others. Indeed, NMR data alone gave conflicting evidence on the relative configuration of the title compound as well. Therefore, an X-ray diffraction experiment was undertaken in order to assess the differences in the stereochemistry with respect to the previously synthesized compounds and the possible mechanistic implications related to the concomitant formation of such stereoisomers in the same reaction.
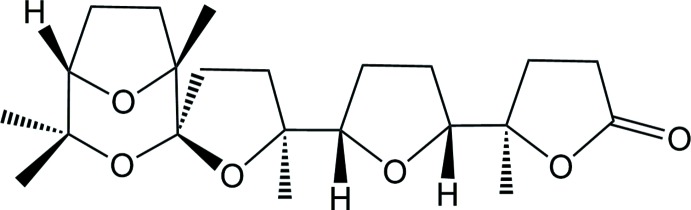


## Structural commentary   

**Figure 3:**
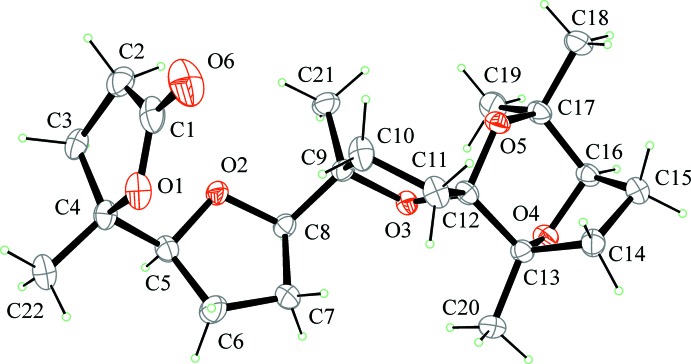
The mol­ecular structure of one of the four crystallographically independent mol­ecules of the title compound (mol­ecule *B*). Displacement ellipsoids are drawn at the 30% probability level.

**Figure 4:**
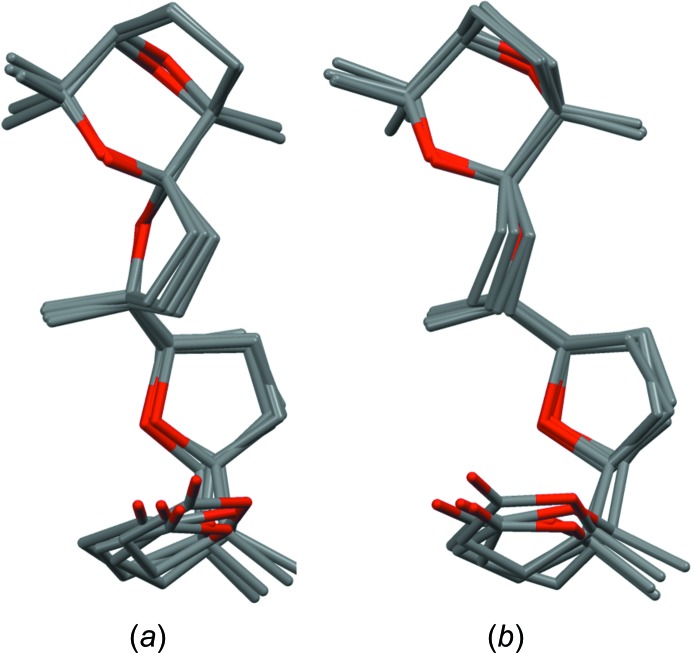
Overlay of the four independent mol­ecules *A*, *B*, *C* and *D* of the title compound viewed in two different orientations (*a*) and (*b*). For mol­ecule *A*, only the major occupancy orientation of the disordered rings is shown.

The crystallographically independent unit contains four mol­ecules of identical configuration. The *ORTEP* diagram of one independent mol­ecule is shown in Fig. 3 ▸. The conformation of the four independent mol­ecules is almost the same, with the exception of the lactone ring, whose orientation is slightly different (Fig. 4 ▸).

**Figure 5:**
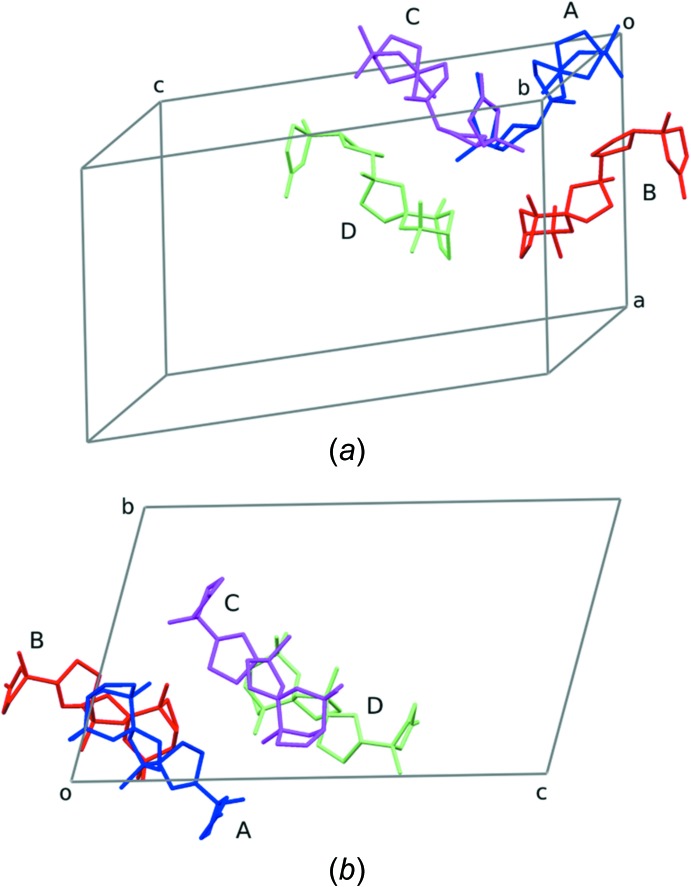
The cluster of the four crystallographic independent mol­ecules of the title compound. (*a*) Skew view; (*b*) view down *a*. For mol­ecule *A*, only the major occupancy orientation of the disordered rings is shown.

The cluster of four independent mol­ecules has approximate local non-crystallographic *C*_2_ symmetry with respect to an axis parallel to *a* and inter­secting the *bc* plane at (*b*/4, *c*/4). This is clearly shown in Fig. 5 ▸. We also note that the pseudo-*C*_2_ symmetry, coupled with truly crystallographic inversion centres, would induce a pseudo-*P*2/*n* symmetry with unique axis *a* (Brock & Dunitz, 1994 ▸).

The presence of more than one formula unit in the asymmetric unit (*Z*′ > 1) can be considered as an ‘exception’ to the normal crystallization behaviour, because only about 12% of the structures archived in the Cambridge Structural Database have *Z*′ > 1 (Brock, 2016 ▸). Actually, the understanding of this phenomenon has been tackled from different points of view. So, structures with *Z*′ > 1 have been considered as the result of ‘mol­ecular association’ (Kitaigorodskii, 1961 ▸), or as ‘frustrated’ crystal structures resulting from competing packing requirements (Anderson *et al.*, 2008 ▸) or as products obtained under kinetic control, *i.e.* ‘fossil relics’ (Steed, 2003 ▸) or ‘crystals on the way’ (Desiraju, 2007 ▸). Actually, one of the problems with high *Z*′ structures is that the apparently most simple and acceptable explanation for their occurrence, *i.e.* that the crystallographic independence comes from the fact that the mol­ecules are related by symmetry operations forbidden in crystals, does not stand. In fact, in many cases of high *Z*′ structures, including the present one, the independent mol­ecules are related to each other by local symmetry operations fully compatible, in principle, with the translational symmetry of the crystals (*i.e.* pseudo inversion centers, pseudo binary axes, *etc*.).

**Figure 6:**
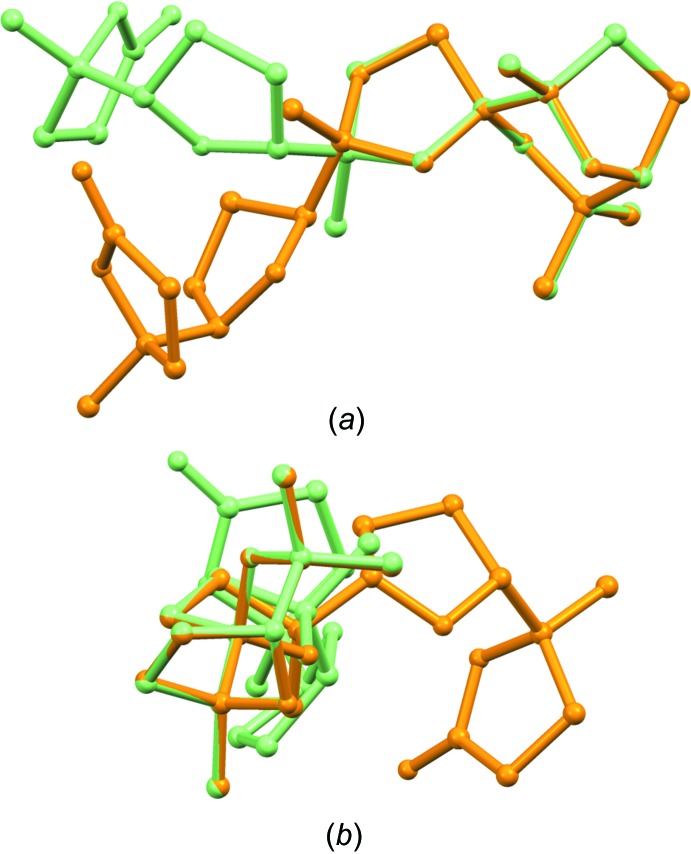
Overlay of mol­ecule *D* of the title compound (green) with mol­ecule *B* of the stereoisomeric compound reported in Piccialli *et al.* (2017 ▸) (orange), in two different orientations, (*a*) and (*b*).

From the analysis of the mol­ecular structure with respect to one previously reported isomeric compound (Piccialli *et al.*, 2017 ▸), it can be seen that the title compound has the same configuration at the stereogenic carbons of the spiro­chetal moiety (C12, C13 and C16), while all of the other four stereogenic carbons (*i.e.* C4, C5, C8, C9) have the opposite configuration, Fig. 6 ▸. This results in a different shape for the two isomers; compared to the previously reported isomer, the title compound has a more horseshoe-type shape. This, in turn, could imply different metal-chelating abilities, that characterize structurally related ionophoric anti­biotics. On the other hand, with respect to the other isomeric compound (compound **10** of Scheme 3 in Piccialli *et al.*, 2009 ▸), the title compound has the opposite configuration only at the C4 and C5 stereogenic carbons.

## Supra­molecular features   

Mol­ecules are held in the crystal basically through van der Waals contacts between H atoms and weak C—H⋯O inter­actions that are detailed in Table 1 ▸.

**Figure 7:**
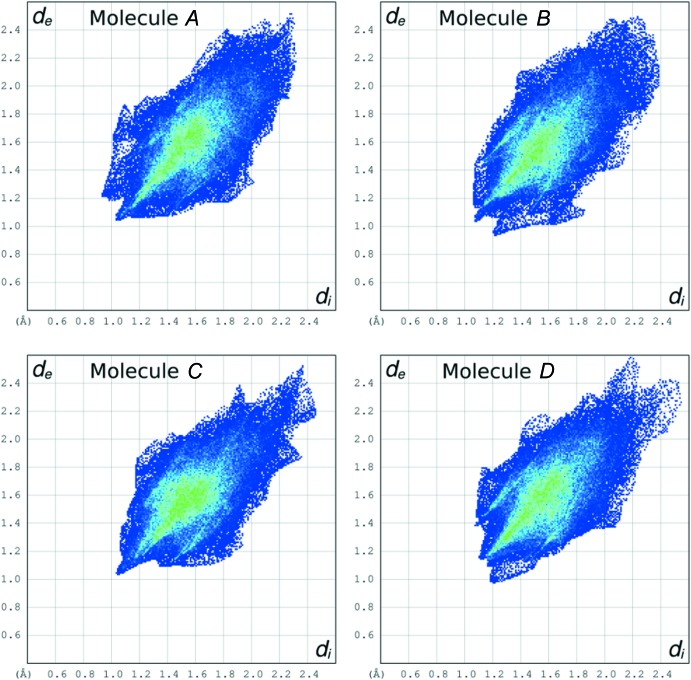
Hirshfeld fingerprint plots of the four crystallographically independent mol­ecules of the title compound.

In order to assess possible packing differences involving the four independent mol­ecules, we have examined their Hirshfeld surfaces (Spackman & McKinnon, 2002 ▸; Wolff *et al.*, 2012 ▸). Fig. 7 ▸ shows the Hirshfeld fingerprint plots of the four independent mol­ecules, while the relevant mol­ecular parameters are reported in Table 2 ▸. In the plots, the distance *d_i_* to the nearest atom inside the surface and the distance *d_e_* to the nearest atom outside the surface are reported for each point of the Hirshfeld surface enveloping the mol­ecule in the crystal. The color of each point in the plot is related to the abundance of that inter­action, from blue (low) to green (high) to red (very high).

A common feature of each plot of Fig. 7 ▸ is represented by the central green stripe, roughly along the diagonal, and centered at *d_i_* + *d_e_* = 3.6 Å. It corresponds to the loose van der Waals contacts present in the packing, and mainly involving H atoms. Another relevant feature is the sting along the diagonal, down to *d_i_* = *d_e_* = 1.0 Å, which reflects points on the Hirshfeld surface that involve nearly head-to-head close H⋯H contacts. This feature is clearly more pronounced in the plots of mol­ecules *A*, *B* and *C*.

## Database survey   

A search of the Cambridge Structural Database (CSD version 5.38, last update May 2017) gave no match for the title compound. A search for spiro-THF compounds gave the same results we have already reported (Piccialli *et al.*, 2017 ▸): six hits (GUHXOX, GUHXUD, MUZTEH, MUZTIL, MUZTOR and MUZTUX) all coming from our research group. The overall fraction of structures deposited in the CSD and having *Z*′ = 4 is 0.48%. This figure drops to 0.24% if the same filters used by Brock (Brock, 2016 ▸) are applied.

## Synthesis and crystallization   

The title compound was prepared by oxidation of squalene with RuO_4_(cat.)/NaIO_4_, as previously reported (Bifulco *et al.*, 2003 ▸). The crude product obtained from the reaction mixture was purified by silica gel column chromatography, eluting with increasing amounts of Et_2_O in hexane. The fractions enriched in the tile compound were collected and evaporated under reduced pressure. Further separation was performed by reversed-phase HPLC (Hibar RP-18 columns, 250 × 10 and 250 × 4 mm, eluent MeOH/H_2_O, 6:4) to give the pure title compound as an oil. Crystals suitable for X-ray diffraction analysis were obtained by slow evaporation of an MeOH solution of the compound.

## Refinement   

Crystal data, data collection and structure refinement details are summarized in Table 3 ▸. The H atoms were generated stereochemically and were refined by the riding model. For all H atoms *U*_iso_ = 1.2×*U*_eq_ of the carrier atom was assumed (1.5 in the case of methyl groups). Some C atoms of two tetra­hydro­furan rings of the independent mol­ecule *A* are disordered over two orientations. The two split positions of the two THF rings were refined by applying DFIX restraints on bond lengths and SIMU restraints on thermal parameters. The final refined occupancy factors of the two components of disorder are 0.694 (9) and 0.306 (9) for one ring and 0.764 (13) and 0.236 (13) for the other.

## Supplementary Material

Crystal structure: contains datablock(s) global, I. DOI: 10.1107/S2056989017010805/zq2238sup1.cif

Structure factors: contains datablock(s) I. DOI: 10.1107/S2056989017010805/zq2238Isup2.hkl

Supporting information file. DOI: 10.1107/S2056989017010805/zq2238Isup3.cml

CCDC reference: 1563862

Additional supporting information:  crystallographic information; 3D view; checkCIF report

## Figures and Tables

**Figure 2:**
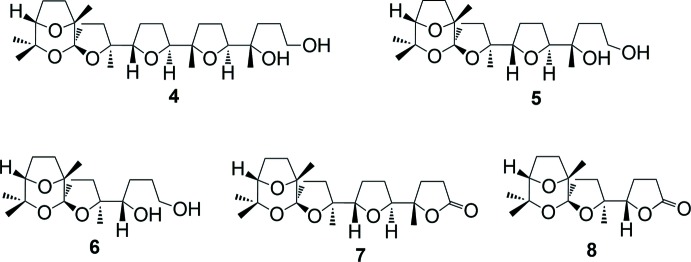
Some small-sized spiro­ketal analogues of compounds **2** and **3** of Fig. 1 ▸.

**Table 1:** Hydrogen-bond geometry (Å, °)

*D*—H⋯*A*	*D*—H	H⋯*A*	*D*⋯*A*	*D*—H⋯*A*
C3*C*—H3*C*1⋯O6*B* ^i^	0.99	2.64	3.300 (4)	125
C22*D*—H22*K*⋯O6*A* ^ii^	0.98	2.55	3.400 (3)	146
C2*B*—H2*B*2⋯O6*D* ^iii^	0.99	2.62	3.423 (4)	139
C22*B*—H22*E*⋯O6*C* ^iv^	0.98	2.68	3.501 (4)	142
C5*B*—H5*B*⋯O4*A*	1.00	2.66	3.510 (3)	143
C2*D*—H2*D*2⋯O6*C* ^v^	0.99	2.67	3.573 (4)	152
C16*D*—H16*D*⋯O1*B* ^i^	1.00	2.61	3.591 (3)	166

Symmetry codes: (i) 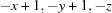; (ii) 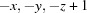; (iii) 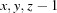; (iv) 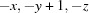; (v) 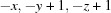.

**Table 2:** Parameters of the Hirshfeld surface (Å^3^, Å^2^) of the four crystallographically independent mol­ecules

Mol­ecule	Volume	Area	Globularity	Asphericity
*A*	524.70	395.49	0.795	0.170
*B*	519.88	392.58	0.796	0.196
*C*	514.41	392.68	0.791	0.181
*D*	524.65	395.99	0.794	0.187

Hirshfeld surface analysis was performed using the program *CrystalExplorer* (Wolff *et al.* 2012 ▸).

**Table 3:** Experimental details

Crystal data	
Chemical formula	C_22_H_34_O_6_
*M*_r_	394.49
Crystal system, space group	Triclinic, *P* 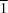
Temperature (K)	173
*a*, *b*, *c* (Å)	13.709 (2), 14.198 (2), 23.339 (2)
α, β, γ (°)	72.878 (10), 82.765 (15), 77.051 (14)
*V* (Å^3^)	4222.2 (11)
*Z*	8
Radiation type	Mo *K*α
μ (mm^−1^)	0.09
Crystal size (mm)	0.50 × 0.37 × 0.25

Data collection	
Diffractometer	Bruker–Nonius KappaCCD
Absorption correction	Multi-scan (*SADABS*; Bruker, 2001 ▸)
*T*_min_, *T*_max_	0.938, 0.958
No. of measured, independent and observed [*I* > 2σ(*I*)] reflections	57303, 19079, 8893
*R*_int_	0.065
(sin θ/λ)_max_ (Å^−1^)	0.650

Refinement	
*R*[*F* ^2^ > 2σ(*F* ^2^)], *wR*(*F* ^2^), *S*	0.067, 0.157, 1.03
No. of reflections	19079
No. of parameters	1058
No. of restraints	52
H-atom treatment	H-atom parameters constrained
Δρ_max_, Δρ_min_ (e Å^−3^)	0.44, −0.33

Computer programs: *COLLECT* (Nonius, 1999 ▸), *DIRAX/LSQ* (Duisenberg *et al.*, 2000 ▸), *EVALCCD* (Duisenberg *et al.*, 2003 ▸), *SIR97* (Altomare *et al.*, 1999 ▸), *SHELXL2016* (Sheldrick, 2015 ▸), *ORTEP-3 for Windows* (Farrugia, 2012 ▸), *Mercury* (Macrae *et al.*, 2006 ▸) and *WinGX* (Farrugia, 2012 ▸).

## supplementary crystallographic information

### Crystal data

**Table:**

C_22_H_34_O_6_	*Z* = 8
*M**_r_* = 394.49	*F*(000) = 1712
Triclinic, *P*1	*D*_x_ = 1.241 Mg m^−^^3^
*a* = 13.709 (2) Å	Mo *K*α radiation, λ = 0.71073 Å
*b* = 14.198 (2) Å	Cell parameters from 132 reflections
*c* = 23.339 (2) Å	θ = 3.0–22.9°
α = 72.878 (10)°	µ = 0.09 mm^−^^1^
β = 82.765 (15)°	*T* = 173 K
γ = 77.051 (14)°	Prism, colourless
*V* = 4222.2 (11) Å^3^	0.50 × 0.37 × 0.25 mm

### Data collection

**Table:**

Bruker–Nonius KappaCCD diffractometer	19079 independent reflections
Radiation source: normal-focus sealed tube	8893 reflections with *I* > 2σ(*I*)
Graphite monochromator	*R*_int_ = 0.065
Detector resolution: 9 pixels mm^-1^	θ_max_ = 27.5°, θ_min_ = 3.0°
CCD rotation images, thick slices scans	*h* = −17→16
Absorption correction: multi-scan (*SADABS*; Bruker, 2001)	*k* = −18→18
*T*_min_ = 0.938, *T*_max_ = 0.958	*l* = −30→25
57303 measured reflections	

### Refinement

**Table:**

Refinement on *F*^2^	Primary atom site location: structure-invariant direct methods
Least-squares matrix: full	Secondary atom site location: difference Fourier map
*R*[*F*^2^ > 2σ(*F*^2^)] = 0.067	Hydrogen site location: inferred from neighbouring sites
*wR*(*F*^2^) = 0.157	H-atom parameters constrained
*S* = 1.03	*w* = 1/[σ^2^(*F*_o_^2^) + (0.052*P*)^2^ + 1.6298*P*] where *P* = (*F*_o_^2^ + 2*F*_c_^2^)/3
19079 reflections	(Δ/σ)_max_ < 0.001
1058 parameters	Δρ_max_ = 0.44 e Å^−^^3^
52 restraints	Δρ_min_ = −0.33 e Å^−^^3^

### Special details

**Table:**

Geometry. All esds (except the esd in the dihedral angle between two l.s. planes) are estimated using the full covariance matrix. The cell esds are taken into account individually in the estimation of esds in distances, angles and torsion angles; correlations between esds in cell parameters are only used when they are defined by crystal symmetry. An approximate (isotropic) treatment of cell esds is used for estimating esds involving l.s. planes.
Refinement. Some C atoms of two tetrahydrofuran rings of the independent molecule A are disordered over two orientations. The two split positions were refined by applying DFIX restraints on bond lengths and SIMU restraints on thermal parameters. The final refined occupancy factors of the two components of disorder are 0.694 (9) and 0.306 (9) for one split position and 0.764 (13) and 0.236 (13) for the other.

### Fractional atomic coordinates and isotropic or equivalent isotropic displacement parameters (Å^2^)

**Table:**

	*x*	*y*	*z*	*U*_iso_*/*U*_eq_	Occ. (<1)
C1A	0.1868 (2)	−0.1356 (2)	0.32673 (12)	0.0385 (7)	
C2A	0.2631 (2)	−0.2133 (2)	0.30641 (14)	0.0530 (9)	
H2A1	0.259973	−0.204806	0.262989	0.064*	
H2A2	0.252890	−0.281628	0.329035	0.064*	
C3A	0.3614 (2)	−0.1965 (2)	0.31902 (14)	0.0496 (8)	
H3A1	0.412875	−0.205398	0.286261	0.060*	
H3A2	0.385264	−0.243833	0.357347	0.060*	
C4A	0.33930 (19)	−0.0889 (2)	0.32283 (12)	0.0354 (7)	
C5A	0.3626 (2)	−0.0131 (2)	0.26344 (12)	0.0374 (7)	
H5A	0.436757	−0.026007	0.253902	0.045*	
C6A	0.3274 (2)	0.0972 (2)	0.26096 (11)	0.0440 (8)	0.306 (9)
H6A1	0.268358	0.106618	0.289304	0.053*	0.306 (9)
H6A2	0.381493	0.124215	0.271191	0.053*	0.306 (9)
C7A	0.3004 (12)	0.1487 (3)	0.1969 (3)	0.0475 (16)	0.306 (9)
H7A1	0.239067	0.201481	0.195408	0.057*	0.306 (9)
H7A2	0.355835	0.179401	0.172874	0.057*	0.306 (9)
C8A	0.28292 (19)	0.06497 (18)	0.17423 (11)	0.0350 (7)	0.306 (9)
H8A	0.337775	0.062960	0.141763	0.042*	0.306 (9)
C6Y	0.3274 (2)	0.0972 (2)	0.26096 (11)	0.0440 (8)	0.694 (9)
H6Y1	0.296235	0.105201	0.300304	0.053*	0.694 (9)
H6Y2	0.383933	0.133295	0.249263	0.053*	0.694 (9)
C7Y	0.2514 (4)	0.1354 (3)	0.2137 (2)	0.0478 (15)	0.694 (9)
H7Y1	0.253742	0.205539	0.190428	0.057*	0.694 (9)
H7Y2	0.182743	0.132266	0.232198	0.057*	0.694 (9)
C8Y	0.28292 (19)	0.06497 (18)	0.17423 (11)	0.0350 (7)	0.694 (9)
H8Y	0.339853	0.085558	0.144014	0.042*	0.694 (9)
C10A	0.1145 (9)	0.0695 (12)	0.1927 (5)	0.0571 (14)	0.236 (13)
H10A	0.082579	0.009999	0.205802	0.069*	0.236 (13)
H10B	0.144030	0.075562	0.227704	0.069*	0.236 (13)
C11A	0.0374 (12)	0.1630 (12)	0.16835 (18)	0.0570 (14)	0.236 (13)
H11A	0.041369	0.217227	0.186110	0.068*	0.236 (13)
H11B	−0.031278	0.148801	0.176677	0.068*	0.236 (13)
C10Y	0.0985 (2)	0.0392 (5)	0.1794 (2)	0.0570 (14)	0.764 (13)
H10W	0.108748	0.014745	0.222946	0.068*	0.764 (13)
H10Z	0.071587	−0.010892	0.167486	0.068*	0.764 (13)
C11Y	0.0283 (4)	0.1404 (5)	0.16451 (15)	0.0569 (14)	0.764 (13)
H11W	0.032847	0.178862	0.192933	0.068*	0.764 (13)
H11Z	−0.041977	0.132859	0.165520	0.068*	0.764 (13)
C9A	0.19546 (18)	0.0575 (2)	0.14357 (12)	0.0434 (8)	
C12A	0.0652 (2)	0.1915 (2)	0.10166 (10)	0.0414 (7)	
C13A	0.0459 (2)	0.3066 (2)	0.07821 (12)	0.0417 (7)	
C14A	−0.0665 (2)	0.3506 (3)	0.07832 (14)	0.0617 (10)	
H14A	−0.103815	0.307985	0.111048	0.074*	
H14B	−0.080387	0.419396	0.083241	0.074*	
C15A	−0.0950 (2)	0.3520 (3)	0.01711 (14)	0.0659 (10)	
H15A	−0.121228	0.421467	−0.006724	0.079*	
H15B	−0.146274	0.310710	0.021317	0.079*	
C16A	0.0033 (2)	0.3071 (2)	−0.01210 (13)	0.0446 (8)	
H16A	0.006303	0.339186	−0.056441	0.054*	
C17A	0.0239 (2)	0.1937 (2)	0.00080 (13)	0.0486 (8)	
C18A	−0.0575 (3)	0.1569 (3)	−0.02122 (17)	0.0789 (12)	
H18A	−0.038742	0.083958	−0.014637	0.118*	
H18B	−0.064244	0.189812	−0.064180	0.118*	
H18C	−0.121453	0.173531	0.001074	0.118*	
C19A	0.1258 (3)	0.1552 (2)	−0.02619 (14)	0.0599 (9)	
H19A	0.177334	0.178654	−0.011499	0.090*	
H19B	0.126693	0.180510	−0.070073	0.090*	
H19C	0.139455	0.081567	−0.014485	0.090*	
C20A	0.1028 (3)	0.3543 (2)	0.10965 (15)	0.0609 (9)	
H20A	0.174683	0.326574	0.105689	0.091*	
H20B	0.080011	0.340221	0.152297	0.091*	
H20C	0.090500	0.427177	0.091374	0.091*	
C21A	0.2313 (4)	−0.0238 (3)	0.10951 (18)	0.0958 (15)	
H21A	0.175728	−0.028151	0.088459	0.144*	
H21B	0.254314	−0.088870	0.138200	0.144*	
H21C	0.286717	−0.005734	0.080305	0.144*	
C22A	0.3893 (3)	−0.0750 (3)	0.37312 (14)	0.0646 (10)	
H22A	0.363696	−0.007061	0.377364	0.097*	
H22B	0.462003	−0.084616	0.363827	0.097*	
H22C	0.374706	−0.124469	0.410773	0.097*	
C1B	0.4756 (2)	0.3466 (3)	−0.18165 (12)	0.0492 (8)	
C2B	0.4330 (2)	0.2585 (2)	−0.18156 (13)	0.0506 (8)	
H2B1	0.454435	0.200366	−0.146885	0.061*	
H2B2	0.454168	0.238327	−0.219103	0.061*	
C3B	0.3201 (2)	0.2961 (2)	−0.17680 (13)	0.0426 (7)	
H3B1	0.286657	0.243797	−0.148792	0.051*	
H3B2	0.292116	0.314855	−0.216616	0.051*	
C4B	0.3061 (2)	0.3875 (2)	−0.15308 (12)	0.0364 (7)	
C5B	0.2899 (2)	0.3621 (2)	−0.08511 (12)	0.0378 (7)	
H5B	0.222272	0.344360	−0.072958	0.045*	
C6B	0.3002 (3)	0.4416 (2)	−0.05548 (14)	0.0597 (10)	
H6B1	0.340607	0.488884	−0.082299	0.072*	
H6B2	0.233413	0.480278	−0.046332	0.072*	
C7B	0.3505 (2)	0.3868 (2)	−0.00005 (14)	0.0532 (9)	
H7B1	0.305090	0.394304	0.035506	0.064*	
H7B2	0.411366	0.412646	0.001109	0.064*	
C8B	0.37856 (19)	0.27638 (19)	−0.00052 (11)	0.0324 (6)	
H8B	0.330605	0.238563	0.027951	0.039*	
C9B	0.48522 (19)	0.22082 (19)	0.01340 (11)	0.0305 (6)	
C10B	0.5671 (2)	0.2792 (2)	−0.01944 (12)	0.0468 (8)	
H10D	0.622555	0.235276	−0.036352	0.056*	
H10E	0.539272	0.337417	−0.052468	0.056*	
C11B	0.6037 (2)	0.3141 (2)	0.02845 (12)	0.0417 (7)	
H11C	0.676326	0.314590	0.021685	0.050*	
H11D	0.566513	0.382138	0.029285	0.050*	
C12B	0.58132 (19)	0.23567 (19)	0.08594 (11)	0.0312 (6)	
C13B	0.56715 (19)	0.26748 (19)	0.14441 (11)	0.0324 (6)	
C14B	0.6639 (2)	0.2933 (2)	0.15682 (13)	0.0421 (7)	
H14C	0.701643	0.321095	0.118835	0.051*	
H14D	0.648925	0.342652	0.180681	0.051*	
C15B	0.7225 (2)	0.1930 (2)	0.19216 (13)	0.0430 (7)	
H15C	0.734307	0.195512	0.232637	0.052*	
H15D	0.787948	0.174521	0.170839	0.052*	
C16B	0.65518 (19)	0.1188 (2)	0.19629 (12)	0.0357 (7)	
H16B	0.661685	0.065476	0.235476	0.043*	
C17B	0.6726 (2)	0.0712 (2)	0.14429 (12)	0.0365 (7)	
C18B	0.7790 (2)	0.0116 (2)	0.13886 (14)	0.0546 (9)	
H18D	0.785557	−0.018108	0.105288	0.082*	
H18E	0.793117	−0.041907	0.176184	0.082*	
H18F	0.826782	0.056560	0.131630	0.082*	
C19B	0.5978 (2)	0.0038 (2)	0.15013 (14)	0.0502 (8)	
H19D	0.529427	0.043207	0.151349	0.075*	
H19E	0.607359	−0.051172	0.187221	0.075*	
H19F	0.608449	−0.024086	0.115585	0.075*	
C20B	0.4746 (2)	0.3481 (2)	0.14775 (14)	0.0492 (8)	
H20D	0.415475	0.326304	0.140509	0.074*	
H20E	0.482502	0.410736	0.117211	0.074*	
H20F	0.466193	0.359419	0.187687	0.074*	
C21B	0.5027 (2)	0.1174 (2)	0.00401 (13)	0.0508 (8)	
H21D	0.570004	0.080800	0.015648	0.076*	
H21E	0.496883	0.123711	−0.038428	0.076*	
H21F	0.452588	0.080718	0.028729	0.076*	
C22B	0.2252 (2)	0.4739 (2)	−0.18264 (13)	0.0527 (9)	
H22D	0.223262	0.531721	−0.167363	0.079*	
H22E	0.160075	0.453431	−0.173431	0.079*	
H22F	0.240083	0.492636	−0.226239	0.079*	
C1C	0.0283 (2)	0.6706 (2)	0.18597 (12)	0.0414 (7)	
C2C	0.0875 (2)	0.7326 (2)	0.20448 (13)	0.0427 (7)	
H2C1	0.083942	0.718580	0.248744	0.051*	
H2C2	0.062465	0.805227	0.186626	0.051*	
C3C	0.1939 (2)	0.7000 (2)	0.18031 (13)	0.0380 (7)	
H3C1	0.243035	0.696850	0.208794	0.046*	
H3C2	0.208888	0.747075	0.141117	0.046*	
C4C	0.19635 (19)	0.5958 (2)	0.17344 (12)	0.0320 (6)	
C5C	0.22903 (19)	0.5119 (2)	0.22866 (12)	0.0338 (7)	
H5C	0.299597	0.511944	0.235443	0.041*	
C6C	0.2222 (2)	0.4072 (2)	0.22795 (14)	0.0534 (9)	
H6C1	0.162670	0.408577	0.207457	0.064*	
H6C2	0.283155	0.375405	0.207796	0.064*	
C7C	0.2130 (2)	0.3527 (2)	0.29386 (14)	0.0571 (9)	
H7C1	0.279982	0.320901	0.309375	0.069*	
H7C2	0.172697	0.300105	0.300832	0.069*	
C8C	0.16093 (19)	0.43333 (19)	0.32405 (12)	0.0332 (7)	
H8C	0.200955	0.428135	0.358226	0.040*	
C9C	0.05220 (19)	0.43183 (19)	0.34820 (12)	0.0325 (6)	
C10C	−0.0189 (2)	0.4321 (2)	0.30422 (14)	0.0555 (9)	
H10F	0.014494	0.442531	0.263312	0.067*	
H10G	−0.078271	0.486899	0.303517	0.067*	
C11C	−0.0501 (2)	0.3319 (2)	0.32427 (12)	0.0448 (8)	
H11E	−0.012089	0.286666	0.300466	0.054*	
H11F	−0.122699	0.339720	0.320292	0.054*	
C12C	−0.02518 (19)	0.29144 (19)	0.38989 (11)	0.0311 (6)	
C13C	0.0059 (2)	0.1767 (2)	0.41399 (11)	0.0359 (7)	
C14C	−0.0818 (2)	0.1254 (2)	0.41442 (13)	0.0468 (8)	
H14E	−0.127127	0.165935	0.382353	0.056*	
H14F	−0.056973	0.057635	0.408538	0.056*	
C15C	−0.1361 (2)	0.1186 (2)	0.47678 (13)	0.0449 (8)	
H15E	−0.207589	0.152163	0.473550	0.054*	
H15F	−0.131831	0.047791	0.500877	0.054*	
C16C	−0.07903 (19)	0.1736 (2)	0.50414 (12)	0.0347 (7)	
H16C	−0.078992	0.144051	0.548684	0.042*	
C17C	−0.11614 (19)	0.28712 (19)	0.48896 (11)	0.0327 (6)	
C18C	−0.0526 (2)	0.3350 (2)	0.51683 (12)	0.0417 (7)	
H18G	0.018312	0.314523	0.504915	0.063*	
H18H	−0.062768	0.313065	0.560702	0.063*	
H18I	−0.072469	0.408385	0.502846	0.063*	
C19C	−0.2261 (2)	0.3160 (2)	0.50934 (14)	0.0487 (8)	
H19G	−0.244050	0.388808	0.503547	0.073*	
H19H	−0.236748	0.281295	0.551955	0.073*	
H19I	−0.268168	0.296348	0.485605	0.073*	
C20C	0.1034 (2)	0.1333 (2)	0.38366 (14)	0.0536 (9)	
H20G	0.156149	0.168023	0.386359	0.080*	
H20H	0.093713	0.142375	0.341334	0.080*	
H20I	0.123066	0.061412	0.403774	0.080*	
C21C	0.0165 (3)	0.5165 (2)	0.37882 (16)	0.0657 (10)	
H21G	−0.050697	0.512393	0.398259	0.099*	
H21H	0.014274	0.581633	0.348699	0.099*	
H21I	0.063009	0.509610	0.409091	0.099*	
C22C	0.2586 (2)	0.5767 (2)	0.11794 (12)	0.0464 (8)	
H22G	0.250160	0.513054	0.112777	0.070*	
H22H	0.329514	0.573183	0.122667	0.070*	
H22I	0.236470	0.631668	0.082561	0.070*	
C1D	0.2932 (2)	0.1876 (2)	0.68680 (12)	0.0398 (7)	
C2D	0.1954 (2)	0.2611 (2)	0.68290 (13)	0.0431 (7)	
H2D1	0.196832	0.319092	0.646896	0.052*	
H2D2	0.180066	0.286005	0.719076	0.052*	
C3D	0.1188 (2)	0.2012 (2)	0.67864 (12)	0.0393 (7)	
H3D1	0.069307	0.242097	0.649058	0.047*	
H3D2	0.082797	0.178370	0.718155	0.047*	
C4D	0.18073 (18)	0.11127 (19)	0.65821 (11)	0.0306 (6)	
C5D	0.18832 (19)	0.12934 (18)	0.59030 (11)	0.0305 (6)	
H5D	0.121489	0.130307	0.576964	0.037*	
C6D	0.2681 (2)	0.0571 (2)	0.56448 (12)	0.0382 (7)	
H6D1	0.327780	0.033252	0.588605	0.046*	
H6D2	0.241797	−0.001727	0.563266	0.046*	
C7D	0.2938 (2)	0.1189 (2)	0.50161 (12)	0.0420 (7)	
H7D1	0.258215	0.104617	0.471680	0.050*	
H7D2	0.366863	0.103822	0.491382	0.050*	
C8D	0.25913 (19)	0.22884 (19)	0.50289 (11)	0.0302 (6)	
H8D	0.205822	0.262273	0.473797	0.036*	
C9D	0.33869 (19)	0.29360 (19)	0.49047 (11)	0.0299 (6)	
C10D	0.43078 (19)	0.2442 (2)	0.52723 (12)	0.0388 (7)	
H10H	0.418163	0.183793	0.559213	0.047*	
H10I	0.448472	0.291782	0.545976	0.047*	
C11D	0.51483 (19)	0.2156 (2)	0.48180 (11)	0.0374 (7)	
H11G	0.580130	0.224380	0.491195	0.045*	
H11H	0.520548	0.145148	0.481240	0.045*	
C12D	0.48164 (18)	0.28854 (19)	0.42232 (11)	0.0296 (6)	
C13D	0.5210 (2)	0.25438 (19)	0.36537 (11)	0.0332 (7)	
C14D	0.6361 (2)	0.2378 (2)	0.35804 (13)	0.0456 (8)	
H14G	0.663718	0.187675	0.335382	0.055*	
H14H	0.665408	0.214440	0.397671	0.055*	
C15D	0.6579 (2)	0.3418 (2)	0.32287 (14)	0.0479 (8)	
H15G	0.696848	0.366585	0.346308	0.058*	
H15H	0.695279	0.339750	0.283966	0.058*	
C16D	0.5536 (2)	0.4073 (2)	0.31351 (12)	0.0405 (7)	
H16D	0.553083	0.458963	0.273669	0.049*	
C17D	0.5108 (2)	0.4578 (2)	0.36347 (13)	0.0404 (7)	
C18D	0.5752 (2)	0.5278 (2)	0.37062 (15)	0.0592 (9)	
H18J	0.543709	0.559902	0.402013	0.089*	
H18K	0.581337	0.579435	0.332535	0.089*	
H18L	0.641975	0.488926	0.381914	0.089*	
C19D	0.4044 (2)	0.5160 (2)	0.35213 (14)	0.0498 (8)	
H19J	0.362463	0.470582	0.348718	0.075*	
H19K	0.404850	0.570259	0.314737	0.075*	
H19L	0.377332	0.544613	0.385615	0.075*	
C20D	0.4788 (2)	0.1671 (2)	0.36103 (13)	0.0440 (8)	
H20J	0.405394	0.183664	0.365222	0.066*	
H20K	0.503446	0.107120	0.393123	0.066*	
H20L	0.500230	0.154165	0.321925	0.066*	
C21D	0.2919 (2)	0.3975 (2)	0.49753 (13)	0.0437 (7)	
H21J	0.343170	0.438942	0.488317	0.066*	
H21K	0.264031	0.391590	0.538935	0.066*	
H21L	0.238161	0.428959	0.469878	0.066*	
C22D	0.1456 (2)	0.0137 (2)	0.68931 (12)	0.0416 (7)	
H22J	0.190698	−0.041602	0.676548	0.062*	
H22K	0.077384	0.018935	0.678559	0.062*	
H22L	0.146088	0.000594	0.732919	0.062*	
O1A	0.23110 (13)	−0.06762 (13)	0.33770 (8)	0.0365 (5)	
O2A	0.31488 (13)	−0.02972 (13)	0.21708 (8)	0.0387 (5)	
O3A	0.17068 (12)	0.15433 (13)	0.09896 (7)	0.0349 (5)	
O4A	0.07830 (14)	0.33320 (13)	0.01515 (8)	0.0403 (5)	
O5A	0.01713 (14)	0.15112 (15)	0.06549 (9)	0.0518 (6)	
O6A	0.09722 (16)	−0.12853 (18)	0.33498 (10)	0.0674 (7)	
O1B	0.40236 (15)	0.42114 (15)	−0.16971 (8)	0.0452 (5)	
O2B	0.36471 (13)	0.27671 (12)	−0.06046 (7)	0.0339 (4)	
O3B	0.49111 (12)	0.21154 (12)	0.07695 (7)	0.0304 (4)	
O4B	0.55606 (13)	0.18001 (13)	0.19357 (8)	0.0367 (5)	
O5B	0.66391 (12)	0.15248 (13)	0.08878 (7)	0.0344 (4)	
O6B	0.56108 (17)	0.3572 (2)	−0.19166 (10)	0.0772 (8)	
O1C	0.09137 (13)	0.59730 (14)	0.16562 (8)	0.0388 (5)	
O2C	0.16517 (13)	0.52856 (12)	0.28005 (7)	0.0331 (4)	
O3C	0.05591 (12)	0.33612 (12)	0.39349 (7)	0.0299 (4)	
O4C	0.02136 (13)	0.15488 (12)	0.47703 (7)	0.0350 (4)	
O5C	−0.11309 (12)	0.32704 (12)	0.42397 (7)	0.0329 (4)	
O6C	−0.06097 (16)	0.68048 (17)	0.18648 (10)	0.0639 (6)	
O1D	0.28161 (12)	0.10150 (14)	0.67671 (8)	0.0352 (5)	
O2D	0.21528 (12)	0.22575 (12)	0.56301 (7)	0.0331 (4)	
O3D	0.37532 (12)	0.30237 (12)	0.42818 (7)	0.0294 (4)	
O4D	0.51448 (13)	0.37898 (13)	0.41994 (8)	0.0368 (5)	
O5D	0.49112 (13)	0.33788 (13)	0.31422 (7)	0.0372 (5)	
O6D	0.37341 (16)	0.19660 (17)	0.69788 (9)	0.0562 (6)	

### Atomic displacement parameters (Å^2^)

**Table:**

	*U*^11^	*U*^22^	*U*^33^	*U*^12^	*U*^13^	*U*^23^
C1A	0.0334 (18)	0.0439 (19)	0.0296 (16)	−0.0088 (15)	−0.0077 (13)	0.0061 (14)
C2A	0.079 (3)	0.0385 (19)	0.0424 (19)	−0.0107 (17)	−0.0058 (17)	−0.0129 (15)
C3A	0.0412 (19)	0.0433 (19)	0.0465 (19)	0.0076 (15)	0.0068 (15)	−0.0010 (15)
C4A	0.0246 (15)	0.0374 (17)	0.0350 (17)	0.0027 (12)	−0.0054 (12)	−0.0014 (13)
C5A	0.0262 (16)	0.0485 (19)	0.0325 (17)	−0.0045 (13)	−0.0077 (12)	−0.0035 (14)
C6A	0.0500 (19)	0.0409 (18)	0.0410 (18)	−0.0148 (14)	−0.0147 (14)	−0.0018 (14)
C7A	0.045 (4)	0.035 (2)	0.067 (3)	−0.012 (3)	−0.035 (3)	−0.005 (2)
C8A	0.0330 (16)	0.0339 (16)	0.0315 (16)	−0.0058 (12)	−0.0050 (13)	0.0017 (13)
C6Y	0.0500 (19)	0.0409 (18)	0.0410 (18)	−0.0148 (14)	−0.0147 (14)	−0.0018 (14)
C7Y	0.046 (3)	0.035 (2)	0.066 (3)	−0.011 (2)	−0.034 (3)	−0.005 (2)
C8Y	0.0330 (16)	0.0339 (16)	0.0315 (16)	−0.0058 (12)	−0.0050 (13)	0.0017 (13)
C10A	0.0392 (18)	0.077 (3)	0.0436 (17)	−0.0289 (16)	−0.0147 (14)	0.0194 (17)
C11A	0.0392 (18)	0.077 (3)	0.0434 (17)	−0.0286 (16)	−0.0144 (14)	0.0190 (17)
C10Y	0.0393 (17)	0.077 (3)	0.0436 (17)	−0.0293 (15)	−0.0149 (13)	0.0196 (16)
C11Y	0.0391 (17)	0.077 (3)	0.0432 (17)	−0.0283 (15)	−0.0141 (13)	0.0187 (16)
C9A	0.0461 (19)	0.0359 (17)	0.0416 (18)	−0.0127 (14)	−0.0150 (14)	0.0078 (15)
C12A	0.0297 (17)	0.058 (2)	0.0289 (16)	−0.0106 (14)	−0.0072 (13)	0.0038 (15)
C13A	0.0407 (18)	0.0478 (19)	0.0301 (17)	−0.0012 (14)	−0.0072 (13)	−0.0044 (14)
C14A	0.046 (2)	0.080 (3)	0.042 (2)	0.0147 (17)	−0.0088 (15)	−0.0080 (18)
C15A	0.051 (2)	0.081 (3)	0.054 (2)	0.0073 (18)	−0.0184 (17)	−0.0082 (19)
C16A	0.049 (2)	0.0465 (19)	0.0328 (17)	−0.0064 (15)	−0.0144 (14)	0.0004 (14)
C17A	0.056 (2)	0.046 (2)	0.0408 (19)	−0.0146 (16)	−0.0194 (16)	0.0014 (15)
C18A	0.098 (3)	0.065 (2)	0.078 (3)	−0.030 (2)	−0.054 (2)	0.004 (2)
C19A	0.087 (3)	0.046 (2)	0.046 (2)	−0.0029 (18)	−0.0196 (18)	−0.0137 (16)
C20A	0.078 (3)	0.048 (2)	0.058 (2)	0.0036 (18)	−0.0280 (18)	−0.0194 (17)
C21A	0.164 (4)	0.041 (2)	0.088 (3)	−0.007 (2)	−0.068 (3)	−0.013 (2)
C22A	0.068 (2)	0.075 (2)	0.044 (2)	−0.0219 (19)	−0.0301 (17)	0.0096 (18)
C1B	0.041 (2)	0.075 (2)	0.0243 (17)	−0.0205 (19)	−0.0005 (14)	0.0033 (16)
C2B	0.046 (2)	0.073 (2)	0.0369 (18)	−0.0143 (17)	0.0040 (14)	−0.0223 (17)
C3B	0.0445 (19)	0.056 (2)	0.0344 (17)	−0.0196 (15)	−0.0058 (13)	−0.0154 (15)
C4B	0.0348 (17)	0.0428 (17)	0.0342 (17)	−0.0168 (14)	−0.0045 (13)	−0.0069 (14)
C5B	0.0363 (17)	0.0389 (17)	0.0357 (17)	−0.0046 (13)	−0.0068 (13)	−0.0066 (14)
C6B	0.092 (3)	0.0417 (19)	0.042 (2)	0.0097 (18)	−0.0229 (18)	−0.0152 (16)
C7B	0.056 (2)	0.0436 (19)	0.061 (2)	0.0117 (15)	−0.0263 (17)	−0.0228 (17)
C8B	0.0378 (17)	0.0336 (16)	0.0258 (15)	−0.0084 (13)	−0.0014 (12)	−0.0072 (12)
C9B	0.0339 (16)	0.0318 (15)	0.0237 (15)	−0.0053 (12)	0.0015 (12)	−0.0068 (12)
C10B	0.0386 (18)	0.069 (2)	0.0283 (17)	−0.0197 (16)	0.0004 (13)	−0.0003 (15)
C11B	0.0363 (17)	0.0444 (18)	0.0392 (18)	−0.0151 (14)	−0.0031 (13)	0.0017 (14)
C12B	0.0257 (15)	0.0306 (15)	0.0339 (16)	−0.0059 (12)	−0.0012 (12)	−0.0039 (13)
C13B	0.0355 (16)	0.0284 (15)	0.0305 (16)	−0.0027 (12)	−0.0036 (12)	−0.0059 (13)
C14B	0.0424 (18)	0.0413 (18)	0.0451 (18)	−0.0069 (14)	−0.0117 (14)	−0.0130 (15)
C15B	0.0383 (18)	0.0451 (18)	0.0437 (18)	−0.0004 (14)	−0.0114 (14)	−0.0117 (15)
C16B	0.0354 (17)	0.0340 (16)	0.0305 (16)	0.0022 (13)	−0.0040 (12)	−0.0038 (13)
C17B	0.0376 (17)	0.0311 (16)	0.0340 (17)	0.0007 (13)	−0.0043 (13)	−0.0033 (13)
C18B	0.051 (2)	0.053 (2)	0.053 (2)	0.0160 (16)	−0.0129 (16)	−0.0182 (17)
C19B	0.063 (2)	0.0321 (17)	0.049 (2)	−0.0045 (15)	−0.0137 (16)	−0.0007 (15)
C20B	0.050 (2)	0.0448 (19)	0.052 (2)	0.0099 (15)	−0.0115 (15)	−0.0225 (16)
C21B	0.069 (2)	0.0379 (18)	0.0423 (19)	0.0086 (15)	−0.0119 (16)	−0.0175 (15)
C22B	0.053 (2)	0.057 (2)	0.0443 (19)	−0.0081 (16)	−0.0203 (15)	−0.0024 (16)
C1C	0.0346 (19)	0.050 (2)	0.0304 (17)	−0.0059 (15)	−0.0018 (14)	0.0009 (15)
C2C	0.0441 (19)	0.0368 (17)	0.0436 (18)	−0.0068 (14)	−0.0006 (14)	−0.0074 (14)
C3C	0.0395 (18)	0.0374 (17)	0.0393 (17)	−0.0136 (13)	−0.0010 (13)	−0.0100 (14)
C4C	0.0268 (15)	0.0397 (17)	0.0330 (16)	−0.0136 (12)	0.0014 (12)	−0.0115 (13)
C5C	0.0264 (15)	0.0382 (17)	0.0339 (16)	−0.0078 (12)	0.0055 (12)	−0.0075 (13)
C6C	0.063 (2)	0.0353 (18)	0.056 (2)	−0.0119 (15)	0.0277 (17)	−0.0155 (16)
C7C	0.049 (2)	0.0345 (18)	0.068 (2)	0.0028 (15)	0.0178 (17)	−0.0013 (17)
C8C	0.0307 (16)	0.0355 (16)	0.0299 (16)	−0.0087 (12)	−0.0056 (12)	−0.0007 (13)
C9C	0.0301 (16)	0.0262 (15)	0.0351 (16)	−0.0044 (12)	−0.0015 (12)	−0.0005 (13)
C10C	0.0436 (19)	0.067 (2)	0.047 (2)	−0.0250 (16)	−0.0222 (15)	0.0174 (17)
C11C	0.058 (2)	0.0448 (19)	0.0329 (17)	−0.0125 (15)	−0.0084 (14)	−0.0084 (14)
C12C	0.0311 (16)	0.0330 (16)	0.0297 (16)	−0.0070 (12)	−0.0042 (12)	−0.0081 (13)
C13C	0.0475 (18)	0.0329 (16)	0.0262 (16)	−0.0072 (13)	0.0020 (13)	−0.0090 (13)
C14C	0.068 (2)	0.0372 (18)	0.0397 (18)	−0.0227 (16)	−0.0023 (15)	−0.0084 (14)
C15C	0.054 (2)	0.0357 (17)	0.0430 (19)	−0.0162 (15)	−0.0030 (15)	−0.0027 (14)
C16C	0.0342 (17)	0.0364 (17)	0.0267 (15)	−0.0038 (13)	0.0031 (12)	−0.0029 (13)
C17C	0.0309 (16)	0.0353 (16)	0.0287 (16)	−0.0056 (12)	0.0009 (12)	−0.0058 (13)
C18C	0.0491 (19)	0.0435 (18)	0.0331 (17)	−0.0088 (14)	0.0019 (13)	−0.0134 (14)
C19C	0.0397 (18)	0.0480 (19)	0.052 (2)	−0.0050 (14)	0.0067 (15)	−0.0102 (16)
C20C	0.071 (2)	0.0324 (17)	0.049 (2)	−0.0006 (16)	0.0126 (17)	−0.0123 (15)
C21C	0.080 (3)	0.0372 (19)	0.070 (2)	−0.0110 (17)	0.0275 (19)	−0.0123 (18)
C22C	0.0497 (19)	0.054 (2)	0.0369 (18)	−0.0201 (15)	0.0136 (14)	−0.0144 (15)
C1D	0.0393 (19)	0.055 (2)	0.0273 (16)	−0.0129 (15)	−0.0019 (13)	−0.0114 (14)
C2D	0.0441 (19)	0.0486 (19)	0.0405 (18)	−0.0070 (15)	−0.0030 (14)	−0.0196 (15)
C3D	0.0324 (17)	0.0464 (18)	0.0352 (17)	0.0005 (14)	−0.0030 (13)	−0.0112 (14)
C4D	0.0232 (15)	0.0360 (16)	0.0291 (16)	−0.0018 (12)	−0.0064 (11)	−0.0048 (13)
C5D	0.0295 (15)	0.0283 (15)	0.0316 (16)	−0.0060 (12)	−0.0070 (12)	−0.0028 (12)
C6D	0.0415 (18)	0.0335 (16)	0.0409 (18)	−0.0077 (13)	−0.0011 (13)	−0.0125 (14)
C7D	0.0481 (19)	0.0395 (18)	0.0404 (18)	−0.0142 (14)	0.0066 (14)	−0.0137 (14)
C8D	0.0318 (16)	0.0335 (16)	0.0239 (15)	−0.0055 (12)	−0.0019 (12)	−0.0068 (12)
C9D	0.0326 (16)	0.0317 (15)	0.0222 (15)	−0.0015 (12)	−0.0033 (11)	−0.0053 (12)
C10D	0.0354 (17)	0.0498 (18)	0.0302 (16)	−0.0062 (14)	−0.0090 (13)	−0.0082 (14)
C11D	0.0306 (16)	0.0411 (17)	0.0350 (17)	−0.0058 (13)	−0.0112 (13)	0.0006 (13)
C12D	0.0271 (16)	0.0297 (15)	0.0301 (16)	−0.0046 (12)	−0.0059 (12)	−0.0044 (12)
C13D	0.0377 (17)	0.0298 (15)	0.0277 (16)	−0.0026 (12)	−0.0053 (12)	−0.0027 (13)
C14D	0.0383 (18)	0.0500 (19)	0.0400 (18)	−0.0009 (14)	0.0024 (14)	−0.0076 (15)
C15D	0.0408 (19)	0.055 (2)	0.0472 (19)	−0.0134 (15)	0.0043 (14)	−0.0131 (16)
C16D	0.0458 (19)	0.0404 (17)	0.0338 (17)	−0.0181 (15)	−0.0030 (13)	−0.0004 (14)
C17D	0.0474 (19)	0.0312 (16)	0.0396 (18)	−0.0126 (14)	−0.0051 (14)	−0.0008 (14)
C18D	0.067 (2)	0.050 (2)	0.067 (2)	−0.0293 (18)	0.0009 (18)	−0.0152 (18)
C19D	0.056 (2)	0.0313 (17)	0.051 (2)	−0.0022 (15)	−0.0039 (16)	0.0006 (15)
C20D	0.054 (2)	0.0362 (17)	0.0419 (18)	−0.0054 (14)	−0.0007 (14)	−0.0149 (15)
C21D	0.052 (2)	0.0385 (18)	0.0402 (18)	−0.0074 (14)	0.0008 (14)	−0.0126 (14)
C22D	0.0400 (18)	0.0423 (18)	0.0371 (17)	−0.0075 (14)	−0.0031 (13)	−0.0029 (14)
O1A	0.0297 (11)	0.0381 (11)	0.0368 (11)	0.0005 (9)	0.0019 (8)	−0.0099 (9)
O2A	0.0457 (12)	0.0323 (11)	0.0314 (11)	0.0019 (9)	−0.0116 (9)	−0.0020 (9)
O3A	0.0309 (11)	0.0348 (11)	0.0329 (11)	−0.0072 (8)	−0.0074 (8)	0.0030 (9)
O4A	0.0459 (12)	0.0387 (11)	0.0320 (11)	−0.0100 (9)	−0.0052 (9)	−0.0009 (9)
O5A	0.0504 (13)	0.0557 (13)	0.0436 (13)	−0.0246 (11)	−0.0220 (10)	0.0132 (10)
O6A	0.0361 (14)	0.0854 (18)	0.0640 (16)	−0.0196 (12)	−0.0130 (11)	0.0144 (13)
O1B	0.0462 (13)	0.0499 (13)	0.0387 (12)	−0.0257 (11)	−0.0087 (10)	0.0033 (10)
O2B	0.0418 (11)	0.0318 (10)	0.0267 (10)	−0.0033 (9)	−0.0064 (8)	−0.0070 (8)
O3B	0.0297 (10)	0.0327 (10)	0.0267 (10)	−0.0075 (8)	−0.0019 (8)	−0.0041 (8)
O4B	0.0345 (11)	0.0405 (11)	0.0292 (11)	−0.0024 (9)	0.0023 (8)	−0.0059 (9)
O5B	0.0305 (11)	0.0365 (11)	0.0295 (11)	0.0018 (8)	0.0010 (8)	−0.0064 (9)
O6B	0.0413 (15)	0.114 (2)	0.0636 (16)	−0.0334 (14)	0.0026 (12)	0.0048 (14)
O1C	0.0365 (12)	0.0531 (13)	0.0313 (11)	−0.0197 (10)	−0.0018 (9)	−0.0102 (10)
O2C	0.0386 (11)	0.0322 (11)	0.0277 (10)	−0.0112 (8)	0.0005 (8)	−0.0051 (9)
O3C	0.0310 (10)	0.0281 (10)	0.0277 (10)	−0.0058 (8)	−0.0036 (8)	−0.0026 (8)
O4C	0.0365 (11)	0.0332 (11)	0.0295 (11)	−0.0005 (8)	0.0011 (8)	−0.0060 (8)
O5C	0.0307 (11)	0.0327 (11)	0.0307 (11)	−0.0037 (8)	−0.0031 (8)	−0.0035 (8)
O6C	0.0324 (13)	0.0878 (18)	0.0617 (15)	−0.0113 (12)	−0.0046 (11)	−0.0053 (13)
O1D	0.0251 (10)	0.0434 (12)	0.0341 (11)	−0.0013 (8)	−0.0085 (8)	−0.0076 (9)
O2D	0.0364 (11)	0.0305 (10)	0.0291 (11)	−0.0044 (8)	0.0009 (8)	−0.0062 (8)
O3D	0.0271 (10)	0.0328 (10)	0.0259 (10)	−0.0029 (8)	−0.0055 (8)	−0.0052 (8)
O4D	0.0427 (12)	0.0372 (11)	0.0325 (11)	−0.0155 (9)	−0.0073 (8)	−0.0047 (9)
O5D	0.0470 (12)	0.0345 (11)	0.0283 (11)	−0.0096 (9)	−0.0072 (9)	−0.0026 (9)
O6D	0.0405 (13)	0.0847 (17)	0.0534 (14)	−0.0198 (12)	−0.0081 (10)	−0.0263 (12)

### Geometric parameters (Å, º)

**Table:**

C1A—O6A	1.205 (3)	C20B—H20E	0.9800
C1A—O1A	1.346 (3)	C20B—H20F	0.9800
C1A—C2A	1.484 (4)	C21B—H21D	0.9800
C2A—C3A	1.498 (4)	C21B—H21E	0.9800
C2A—H2A1	0.9900	C21B—H21F	0.9800
C2A—H2A2	0.9900	C22B—H22D	0.9800
C3A—C4A	1.516 (4)	C22B—H22E	0.9800
C3A—H3A1	0.9900	C22B—H22F	0.9800
C3A—H3A2	0.9900	C1C—O6C	1.198 (3)
C4A—O1A	1.464 (3)	C1C—O1C	1.356 (3)
C4A—C22A	1.512 (4)	C1C—C2C	1.501 (4)
C4A—C5A	1.532 (4)	C2C—C3C	1.517 (4)
C5A—O2A	1.433 (3)	C2C—H2C1	0.9900
C5A—C6Y	1.517 (4)	C2C—H2C2	0.9900
C5A—C6A	1.517 (4)	C3C—C4C	1.527 (4)
C5A—H5A	1.0000	C3C—H3C1	0.9900
C6A—C7A	1.5100 (10)	C3C—H3C2	0.9900
C6A—H6A1	0.9900	C4C—O1C	1.468 (3)
C6A—H6A2	0.9900	C4C—C5C	1.511 (4)
C7A—C8A	1.5099 (10)	C4C—C22C	1.518 (4)
C7A—H7A1	0.9900	C5C—O2C	1.439 (3)
C7A—H7A2	0.9900	C5C—C6C	1.515 (4)
C8A—O2A	1.433 (3)	C5C—H5C	1.0000
C8A—C9A	1.509 (4)	C6C—C7C	1.508 (4)
C8A—H8A	1.0000	C6C—H6C1	0.9900
C6Y—C7Y	1.5095 (10)	C6C—H6C2	0.9900
C6Y—H6Y1	0.9900	C7C—C8C	1.516 (4)
C6Y—H6Y2	0.9900	C7C—H7C1	0.9900
C7Y—C8Y	1.5112 (10)	C7C—H7C2	0.9900
C7Y—H7Y1	0.9900	C8C—O2C	1.445 (3)
C7Y—H7Y2	0.9900	C8C—C9C	1.528 (3)
C8Y—O2A	1.433 (3)	C8C—H8C	1.0000
C8Y—C9A	1.509 (4)	C9C—O3C	1.450 (3)
C8Y—H8Y	1.0169	C9C—C10C	1.501 (4)
C10A—C11A	1.5101 (11)	C9C—C21C	1.533 (4)
C10A—C9A	1.5108 (11)	C10C—C11C	1.501 (4)
C10A—H10A	0.9900	C10C—H10F	0.9900
C10A—H10B	0.9900	C10C—H10G	0.9900
C11A—C12A	1.5101 (10)	C11C—C12C	1.522 (4)
C11A—H11A	0.9900	C11C—H11E	0.9900
C11A—H11B	0.9900	C11C—H11F	0.9900
C10Y—C9A	1.5097 (10)	C12C—O3C	1.420 (3)
C10Y—C11Y	1.5108 (11)	C12C—O5C	1.436 (3)
C10Y—H10W	0.9900	C12C—C13C	1.537 (4)
C10Y—H10Z	0.9900	C13C—O4C	1.444 (3)
C11Y—C12A	1.5112 (10)	C13C—C20C	1.522 (4)
C11Y—H11W	0.9900	C13C—C14C	1.537 (4)
C11Y—H11Z	0.9900	C14C—C15C	1.536 (4)
C9A—O3A	1.458 (3)	C14C—H14E	0.9900
C9A—C21A	1.547 (5)	C14C—H14F	0.9900
C12A—O3A	1.424 (3)	C15C—C16C	1.524 (4)
C12A—O5A	1.439 (3)	C15C—H15E	0.9900
C12A—C13A	1.535 (4)	C15C—H15F	0.9900
C13A—O4A	1.445 (3)	C16C—O4C	1.443 (3)
C13A—C20A	1.510 (4)	C16C—C17C	1.524 (4)
C13A—C14A	1.527 (4)	C16C—H16C	1.0000
C14A—C15A	1.522 (4)	C17C—O5C	1.453 (3)
C14A—H14A	0.9900	C17C—C18C	1.521 (4)
C14A—H14B	0.9900	C17C—C19C	1.524 (4)
C15A—C16A	1.525 (4)	C18C—H18G	0.9800
C15A—H15A	0.9900	C18C—H18H	0.9800
C15A—H15B	0.9900	C18C—H18I	0.9800
C16A—O4A	1.440 (3)	C19C—H19G	0.9800
C16A—C17A	1.515 (4)	C19C—H19H	0.9800
C16A—H16A	1.0000	C19C—H19I	0.9800
C17A—O5A	1.451 (3)	C20C—H20G	0.9800
C17A—C19A	1.512 (4)	C20C—H20H	0.9800
C17A—C18A	1.531 (4)	C20C—H20I	0.9800
C18A—H18A	0.9800	C21C—H21G	0.9800
C18A—H18B	0.9800	C21C—H21H	0.9800
C18A—H18C	0.9800	C21C—H21I	0.9800
C19A—H19A	0.9800	C22C—H22G	0.9800
C19A—H19B	0.9800	C22C—H22H	0.9800
C19A—H19C	0.9800	C22C—H22I	0.9800
C20A—H20A	0.9800	C1D—O6D	1.203 (3)
C20A—H20B	0.9800	C1D—O1D	1.357 (3)
C20A—H20C	0.9800	C1D—C2D	1.497 (4)
C21A—H21A	0.9800	C2D—C3D	1.521 (4)
C21A—H21B	0.9800	C2D—H2D1	0.9900
C21A—H21C	0.9800	C2D—H2D2	0.9900
C22A—H22A	0.9800	C3D—C4D	1.532 (4)
C22A—H22B	0.9800	C3D—H3D1	0.9900
C22A—H22C	0.9800	C3D—H3D2	0.9900
C1B—O6B	1.200 (3)	C4D—O1D	1.465 (3)
C1B—O1B	1.354 (4)	C4D—C22D	1.516 (4)
C1B—C2B	1.494 (4)	C4D—C5D	1.523 (3)
C2B—C3B	1.520 (4)	C5D—O2D	1.439 (3)
C2B—H2B1	0.9900	C5D—C6D	1.519 (4)
C2B—H2B2	0.9900	C5D—H5D	1.0000
C3B—C4B	1.521 (4)	C6D—C7D	1.515 (4)
C3B—H3B1	0.9900	C6D—H6D1	0.9900
C3B—H3B2	0.9900	C6D—H6D2	0.9900
C4B—O1B	1.472 (3)	C7D—C8D	1.535 (4)
C4B—C22B	1.515 (4)	C7D—H7D1	0.9900
C4B—C5B	1.519 (4)	C7D—H7D2	0.9900
C5B—O2B	1.426 (3)	C8D—O2D	1.448 (3)
C5B—C6B	1.526 (4)	C8D—C9D	1.527 (3)
C5B—H5B	1.0000	C8D—H8D	1.0000
C6B—C7B	1.465 (4)	C9D—O3D	1.456 (3)
C6B—H6B1	0.9900	C9D—C21D	1.519 (4)
C6B—H6B2	0.9900	C9D—C10D	1.531 (3)
C7B—C8B	1.531 (4)	C10D—C11D	1.534 (4)
C7B—H7B1	0.9900	C10D—H10H	0.9900
C7B—H7B2	0.9900	C10D—H10I	0.9900
C8B—O2B	1.434 (3)	C11D—C12D	1.519 (3)
C8B—C9B	1.522 (3)	C11D—H11G	0.9900
C8B—H8B	1.0000	C11D—H11H	0.9900
C9B—O3B	1.461 (3)	C12D—O3D	1.421 (3)
C9B—C21B	1.509 (4)	C12D—O4D	1.437 (3)
C9B—C10B	1.536 (4)	C12D—C13D	1.541 (4)
C10B—C11B	1.525 (4)	C13D—O5D	1.439 (3)
C10B—H10D	0.9900	C13D—C20D	1.513 (4)
C10B—H10E	0.9900	C13D—C14D	1.537 (4)
C11B—C12B	1.518 (4)	C14D—C15D	1.538 (4)
C11B—H11C	0.9900	C14D—H14G	0.9900
C11B—H11D	0.9900	C14D—H14H	0.9900
C12B—O3B	1.410 (3)	C15D—C16D	1.522 (4)
C12B—O5B	1.433 (3)	C15D—H15G	0.9900
C12B—C13B	1.536 (4)	C15D—H15H	0.9900
C13B—O4B	1.443 (3)	C16D—O5D	1.440 (3)
C13B—C20B	1.517 (4)	C16D—C17D	1.533 (4)
C13B—C14B	1.534 (4)	C16D—H16D	1.0000
C14B—C15B	1.522 (4)	C17D—O4D	1.454 (3)
C14B—H14C	0.9900	C17D—C19D	1.519 (4)
C14B—H14D	0.9900	C17D—C18D	1.524 (4)
C15B—C16B	1.525 (4)	C18D—H18J	0.9800
C15B—H15C	0.9900	C18D—H18K	0.9800
C15B—H15D	0.9900	C18D—H18L	0.9800
C16B—O4B	1.437 (3)	C19D—H19J	0.9800
C16B—C17B	1.524 (4)	C19D—H19K	0.9800
C16B—H16B	1.0000	C19D—H19L	0.9800
C17B—O5B	1.457 (3)	C20D—H20J	0.9800
C17B—C19B	1.522 (4)	C20D—H20K	0.9800
C17B—C18B	1.524 (4)	C20D—H20L	0.9800
C18B—H18D	0.9800	C21D—H21J	0.9800
C18B—H18E	0.9800	C21D—H21K	0.9800
C18B—H18F	0.9800	C21D—H21L	0.9800
C19B—H19D	0.9800	C22D—H22J	0.9800
C19B—H19E	0.9800	C22D—H22K	0.9800
C19B—H19F	0.9800	C22D—H22L	0.9800
C20B—H20D	0.9800		
			
O6A—C1A—O1A	120.1 (3)	H21D—C21B—H21F	109.5
O6A—C1A—C2A	129.5 (3)	H21E—C21B—H21F	109.5
O1A—C1A—C2A	110.4 (2)	C4B—C22B—H22D	109.5
C1A—C2A—C3A	104.5 (2)	C4B—C22B—H22E	109.5
C1A—C2A—H2A1	110.8	H22D—C22B—H22E	109.5
C3A—C2A—H2A1	110.8	C4B—C22B—H22F	109.5
C1A—C2A—H2A2	110.8	H22D—C22B—H22F	109.5
C3A—C2A—H2A2	110.8	H22E—C22B—H22F	109.5
H2A1—C2A—H2A2	108.9	O6C—C1C—O1C	121.4 (3)
C2A—C3A—C4A	104.5 (2)	O6C—C1C—C2C	128.7 (3)
C2A—C3A—H3A1	110.8	O1C—C1C—C2C	109.9 (2)
C4A—C3A—H3A1	110.8	C1C—C2C—C3C	104.1 (2)
C2A—C3A—H3A2	110.8	C1C—C2C—H2C1	110.9
C4A—C3A—H3A2	110.8	C3C—C2C—H2C1	110.9
H3A1—C3A—H3A2	108.9	C1C—C2C—H2C2	110.9
O1A—C4A—C22A	106.6 (2)	C3C—C2C—H2C2	110.9
O1A—C4A—C3A	104.3 (2)	H2C1—C2C—H2C2	109.0
C22A—C4A—C3A	113.5 (2)	C2C—C3C—C4C	104.3 (2)
O1A—C4A—C5A	108.36 (19)	C2C—C3C—H3C1	110.9
C22A—C4A—C5A	110.1 (2)	C4C—C3C—H3C1	110.9
C3A—C4A—C5A	113.4 (2)	C2C—C3C—H3C2	110.9
O2A—C5A—C6Y	106.56 (19)	C4C—C3C—H3C2	110.9
O2A—C5A—C6A	106.56 (19)	H3C1—C3C—H3C2	108.9
O2A—C5A—C4A	108.2 (2)	O1C—C4C—C5C	108.15 (19)
C6Y—C5A—C4A	116.4 (2)	O1C—C4C—C22C	107.9 (2)
C6A—C5A—C4A	116.4 (2)	C5C—C4C—C22C	110.5 (2)
O2A—C5A—H5A	108.5	O1C—C4C—C3C	103.7 (2)
C6A—C5A—H5A	108.5	C5C—C4C—C3C	113.1 (2)
C4A—C5A—H5A	108.5	C22C—C4C—C3C	113.0 (2)
C7A—C6A—C5A	105.0 (3)	O2C—C5C—C4C	109.4 (2)
C7A—C6A—H6A1	110.7	O2C—C5C—C6C	104.4 (2)
C5A—C6A—H6A1	110.7	C4C—C5C—C6C	116.1 (2)
C7A—C6A—H6A2	110.7	O2C—C5C—H5C	108.9
C5A—C6A—H6A2	110.7	C4C—C5C—H5C	108.9
H6A1—C6A—H6A2	108.8	C6C—C5C—H5C	108.9
C8A—C7A—C6A	104.0 (2)	C7C—C6C—C5C	102.8 (2)
C8A—C7A—H7A1	111.0	C7C—C6C—H6C1	111.2
C6A—C7A—H7A1	111.0	C5C—C6C—H6C1	111.2
C8A—C7A—H7A2	111.0	C7C—C6C—H6C2	111.2
C6A—C7A—H7A2	111.0	C5C—C6C—H6C2	111.2
H7A1—C7A—H7A2	109.0	H6C1—C6C—H6C2	109.1
O2A—C8A—C9A	108.5 (2)	C6C—C7C—C8C	104.9 (2)
O2A—C8A—C7A	109.4 (2)	C6C—C7C—H7C1	110.8
C9A—C8A—C7A	130.7 (5)	C8C—C7C—H7C1	110.8
O2A—C8A—H8A	101.1	C6C—C7C—H7C2	110.8
C9A—C8A—H8A	101.1	C8C—C7C—H7C2	110.8
C7A—C8A—H8A	101.4	H7C1—C7C—H7C2	108.8
C7Y—C6Y—C5A	103.9 (2)	O2C—C8C—C7C	106.2 (2)
C7Y—C6Y—H6Y1	111.0	O2C—C8C—C9C	108.98 (19)
C5A—C6Y—H6Y1	111.0	C7C—C8C—C9C	117.2 (2)
C7Y—C6Y—H6Y2	111.0	O2C—C8C—H8C	108.0
C5A—C6Y—H6Y2	111.0	C7C—C8C—H8C	108.0
H6Y1—C6Y—H6Y2	109.0	C9C—C8C—H8C	108.0
C6Y—C7Y—C8Y	103.9 (2)	O3C—C9C—C10C	105.1 (2)
C6Y—C7Y—H7Y1	111.0	O3C—C9C—C8C	105.05 (19)
C8Y—C7Y—H7Y1	111.0	C10C—C9C—C8C	116.1 (2)
C6Y—C7Y—H7Y2	111.0	O3C—C9C—C21C	108.7 (2)
C8Y—C7Y—H7Y2	111.0	C10C—C9C—C21C	113.1 (3)
H7Y1—C7Y—H7Y2	109.0	C8C—C9C—C21C	108.3 (2)
O2A—C8Y—C9A	108.5 (2)	C9C—C10C—C11C	107.3 (2)
O2A—C8Y—C7Y	102.2 (2)	C9C—C10C—H10F	110.3
C9A—C8Y—C7Y	111.8 (2)	C11C—C10C—H10F	110.3
O2A—C8Y—H8Y	111.7	C9C—C10C—H10G	110.3
C9A—C8Y—H8Y	111.5	C11C—C10C—H10G	110.3
C7Y—C8Y—H8Y	110.9	H10F—C10C—H10G	108.5
C11A—C10A—C9A	108.5 (8)	C10C—C11C—C12C	104.0 (2)
C11A—C10A—H10A	110.0	C10C—C11C—H11E	111.0
C9A—C10A—H10A	110.0	C12C—C11C—H11E	111.0
C11A—C10A—H10B	110.0	C10C—C11C—H11F	111.0
C9A—C10A—H10B	110.0	C12C—C11C—H11F	111.0
H10A—C10A—H10B	108.4	H11E—C11C—H11F	109.0
C10A—C11A—C12A	103.6 (8)	O3C—C12C—O5C	111.2 (2)
C10A—C11A—H11A	111.0	O3C—C12C—C11C	104.4 (2)
C12A—C11A—H11A	111.0	O5C—C12C—C11C	106.1 (2)
C10A—C11A—H11B	111.0	O3C—C12C—C13C	109.0 (2)
C12A—C11A—H11B	111.0	O5C—C12C—C13C	109.6 (2)
H11A—C11A—H11B	109.0	C11C—C12C—C13C	116.4 (2)
C9A—C10Y—C11Y	104.7 (3)	O4C—C13C—C20C	107.5 (2)
C9A—C10Y—H10W	110.8	O4C—C13C—C12C	107.1 (2)
C11Y—C10Y—H10W	110.8	C20C—C13C—C12C	113.3 (2)
C9A—C10Y—H10Z	110.8	O4C—C13C—C14C	103.1 (2)
C11Y—C10Y—H10Z	110.8	C20C—C13C—C14C	114.2 (2)
H10W—C10Y—H10Z	108.9	C12C—C13C—C14C	110.8 (2)
C10Y—C11Y—C12A	103.4 (3)	C15C—C14C—C13C	104.6 (2)
C10Y—C11Y—H11W	111.1	C15C—C14C—H14E	110.8
C12A—C11Y—H11W	111.1	C13C—C14C—H14E	110.8
C10Y—C11Y—H11Z	111.1	C15C—C14C—H14F	110.8
C12A—C11Y—H11Z	111.1	C13C—C14C—H14F	110.8
H11W—C11Y—H11Z	109.0	H14E—C14C—H14F	108.9
O3A—C9A—C8A	105.2 (2)	C16C—C15C—C14C	102.9 (2)
O3A—C9A—C8Y	105.2 (2)	C16C—C15C—H15E	111.2
O3A—C9A—C10Y	106.1 (2)	C14C—C15C—H15E	111.2
O3A—C9A—C10A	101.2 (6)	C16C—C15C—H15F	111.2
O3A—C9A—C21A	107.7 (2)	C14C—C15C—H15F	111.2
C8A—C9A—C21A	108.5 (2)	H15E—C15C—H15F	109.1
C8Y—C9A—C21A	108.5 (2)	O4C—C16C—C15C	103.5 (2)
C10Y—C9A—C21A	108.5 (4)	O4C—C16C—C17C	107.7 (2)
C10A—C9A—C21A	133.0 (8)	C15C—C16C—C17C	114.8 (2)
O3A—C12A—O5A	110.3 (2)	O4C—C16C—H16C	110.2
O3A—C12A—C11A	102.6 (6)	C15C—C16C—H16C	110.2
O5A—C12A—C11A	116.6 (8)	C17C—C16C—H16C	110.2
O3A—C12A—C11Y	105.1 (2)	O5C—C17C—C18C	111.4 (2)
O5A—C12A—C11Y	102.0 (3)	O5C—C17C—C19C	104.5 (2)
O3A—C12A—C13A	108.5 (2)	C18C—C17C—C19C	109.5 (2)
O5A—C12A—C13A	109.8 (2)	O5C—C17C—C16C	108.3 (2)
C11A—C12A—C13A	108.6 (7)	C18C—C17C—C16C	110.6 (2)
C11Y—C12A—C13A	120.8 (3)	C19C—C17C—C16C	112.4 (2)
O4A—C13A—C20A	107.6 (2)	C17C—C18C—H18G	109.5
O4A—C13A—C14A	103.2 (2)	C17C—C18C—H18H	109.5
C20A—C13A—C14A	113.1 (3)	H18G—C18C—H18H	109.5
O4A—C13A—C12A	108.3 (2)	C17C—C18C—H18I	109.5
C20A—C13A—C12A	113.1 (2)	H18G—C18C—H18I	109.5
C14A—C13A—C12A	110.9 (2)	H18H—C18C—H18I	109.5
C15A—C14A—C13A	104.0 (2)	C17C—C19C—H19G	109.5
C15A—C14A—H14A	111.0	C17C—C19C—H19H	109.5
C13A—C14A—H14A	111.0	H19G—C19C—H19H	109.5
C15A—C14A—H14B	111.0	C17C—C19C—H19I	109.5
C13A—C14A—H14B	111.0	H19G—C19C—H19I	109.5
H14A—C14A—H14B	109.0	H19H—C19C—H19I	109.5
C14A—C15A—C16A	103.7 (2)	C13C—C20C—H20G	109.5
C14A—C15A—H15A	111.0	C13C—C20C—H20H	109.5
C16A—C15A—H15A	111.0	H20G—C20C—H20H	109.5
C14A—C15A—H15B	111.0	C13C—C20C—H20I	109.5
C16A—C15A—H15B	111.0	H20G—C20C—H20I	109.5
H15A—C15A—H15B	109.0	H20H—C20C—H20I	109.5
O4A—C16A—C17A	107.8 (2)	C9C—C21C—H21G	109.5
O4A—C16A—C15A	103.4 (2)	C9C—C21C—H21H	109.5
C17A—C16A—C15A	114.8 (3)	H21G—C21C—H21H	109.5
O4A—C16A—H16A	110.2	C9C—C21C—H21I	109.5
C17A—C16A—H16A	110.2	H21G—C21C—H21I	109.5
C15A—C16A—H16A	110.2	H21H—C21C—H21I	109.5
O5A—C17A—C19A	111.0 (2)	C4C—C22C—H22G	109.5
O5A—C17A—C16A	108.0 (2)	C4C—C22C—H22H	109.5
C19A—C17A—C16A	111.2 (3)	H22G—C22C—H22H	109.5
O5A—C17A—C18A	104.1 (2)	C4C—C22C—H22I	109.5
C19A—C17A—C18A	110.2 (3)	H22G—C22C—H22I	109.5
C16A—C17A—C18A	112.2 (2)	H22H—C22C—H22I	109.5
C17A—C18A—H18A	109.5	O6D—C1D—O1D	120.8 (3)
C17A—C18A—H18B	109.5	O6D—C1D—C2D	128.7 (3)
H18A—C18A—H18B	109.5	O1D—C1D—C2D	110.5 (2)
C17A—C18A—H18C	109.5	C1D—C2D—C3D	104.2 (2)
H18A—C18A—H18C	109.5	C1D—C2D—H2D1	110.9
H18B—C18A—H18C	109.5	C3D—C2D—H2D1	110.9
C17A—C19A—H19A	109.5	C1D—C2D—H2D2	110.9
C17A—C19A—H19B	109.5	C3D—C2D—H2D2	110.9
H19A—C19A—H19B	109.5	H2D1—C2D—H2D2	108.9
C17A—C19A—H19C	109.5	C2D—C3D—C4D	104.7 (2)
H19A—C19A—H19C	109.5	C2D—C3D—H3D1	110.8
H19B—C19A—H19C	109.5	C4D—C3D—H3D1	110.8
C13A—C20A—H20A	109.5	C2D—C3D—H3D2	110.8
C13A—C20A—H20B	109.5	C4D—C3D—H3D2	110.8
H20A—C20A—H20B	109.5	H3D1—C3D—H3D2	108.9
C13A—C20A—H20C	109.5	O1D—C4D—C22D	107.83 (19)
H20A—C20A—H20C	109.5	O1D—C4D—C5D	107.55 (19)
H20B—C20A—H20C	109.5	C22D—C4D—C5D	110.5 (2)
C9A—C21A—H21A	109.5	O1D—C4D—C3D	104.0 (2)
C9A—C21A—H21B	109.5	C22D—C4D—C3D	113.2 (2)
H21A—C21A—H21B	109.5	C5D—C4D—C3D	113.2 (2)
C9A—C21A—H21C	109.5	O2D—C5D—C6D	104.1 (2)
H21A—C21A—H21C	109.5	O2D—C5D—C4D	108.2 (2)
H21B—C21A—H21C	109.5	C6D—C5D—C4D	117.0 (2)
C4A—C22A—H22A	109.5	O2D—C5D—H5D	109.1
C4A—C22A—H22B	109.5	C6D—C5D—H5D	109.1
H22A—C22A—H22B	109.5	C4D—C5D—H5D	109.1
C4A—C22A—H22C	109.5	C7D—C6D—C5D	104.1 (2)
H22A—C22A—H22C	109.5	C7D—C6D—H6D1	110.9
H22B—C22A—H22C	109.5	C5D—C6D—H6D1	110.9
O6B—C1B—O1B	120.6 (3)	C7D—C6D—H6D2	110.9
O6B—C1B—C2B	128.6 (3)	C5D—C6D—H6D2	110.9
O1B—C1B—C2B	110.8 (3)	H6D1—C6D—H6D2	108.9
C1B—C2B—C3B	104.0 (3)	C6D—C7D—C8D	105.2 (2)
C1B—C2B—H2B1	111.0	C6D—C7D—H7D1	110.7
C3B—C2B—H2B1	111.0	C8D—C7D—H7D1	110.7
C1B—C2B—H2B2	111.0	C6D—C7D—H7D2	110.7
C3B—C2B—H2B2	111.0	C8D—C7D—H7D2	110.7
H2B1—C2B—H2B2	109.0	H7D1—C7D—H7D2	108.8
C2B—C3B—C4B	105.0 (2)	O2D—C8D—C9D	106.81 (19)
C2B—C3B—H3B1	110.7	O2D—C8D—C7D	105.97 (19)
C4B—C3B—H3B1	110.7	C9D—C8D—C7D	117.7 (2)
C2B—C3B—H3B2	110.7	O2D—C8D—H8D	108.7
C4B—C3B—H3B2	110.7	C9D—C8D—H8D	108.7
H3B1—C3B—H3B2	108.8	C7D—C8D—H8D	108.7
O1B—C4B—C22B	107.3 (2)	O3D—C9D—C21D	109.2 (2)
O1B—C4B—C5B	107.5 (2)	O3D—C9D—C8D	105.53 (19)
C22B—C4B—C5B	110.8 (2)	C21D—C9D—C8D	110.2 (2)
O1B—C4B—C3B	104.1 (2)	O3D—C9D—C10D	105.35 (19)
C22B—C4B—C3B	113.7 (2)	C21D—C9D—C10D	112.5 (2)
C5B—C4B—C3B	112.9 (2)	C8D—C9D—C10D	113.6 (2)
O2B—C5B—C4B	107.8 (2)	C9D—C10D—C11D	104.9 (2)
O2B—C5B—C6B	104.9 (2)	C9D—C10D—H10H	110.8
C4B—C5B—C6B	117.1 (2)	C11D—C10D—H10H	110.8
O2B—C5B—H5B	108.9	C9D—C10D—H10I	110.8
C4B—C5B—H5B	108.9	C11D—C10D—H10I	110.8
C6B—C5B—H5B	108.9	H10H—C10D—H10I	108.8
C7B—C6B—C5B	106.0 (2)	C12D—C11D—C10D	103.4 (2)
C7B—C6B—H6B1	110.5	C12D—C11D—H11G	111.1
C5B—C6B—H6B1	110.5	C10D—C11D—H11G	111.1
C7B—C6B—H6B2	110.5	C12D—C11D—H11H	111.1
C5B—C6B—H6B2	110.5	C10D—C11D—H11H	111.1
H6B1—C6B—H6B2	108.7	H11G—C11D—H11H	109.1
C6B—C7B—C8B	106.3 (2)	O3D—C12D—O4D	111.39 (19)
C6B—C7B—H7B1	110.5	O3D—C12D—C11D	104.13 (19)
C8B—C7B—H7B1	110.5	O4D—C12D—C11D	104.8 (2)
C6B—C7B—H7B2	110.5	O3D—C12D—C13D	109.34 (19)
C8B—C7B—H7B2	110.5	O4D—C12D—C13D	110.4 (2)
H7B1—C7B—H7B2	108.7	C11D—C12D—C13D	116.6 (2)
O2B—C8B—C9B	107.1 (2)	O5D—C13D—C20D	107.6 (2)
O2B—C8B—C7B	105.3 (2)	O5D—C13D—C14D	102.7 (2)
C9B—C8B—C7B	117.6 (2)	C20D—C13D—C14D	114.1 (2)
O2B—C8B—H8B	108.8	O5D—C13D—C12D	107.6 (2)
C9B—C8B—H8B	108.8	C20D—C13D—C12D	113.4 (2)
C7B—C8B—H8B	108.8	C14D—C13D—C12D	110.6 (2)
O3B—C9B—C21B	109.5 (2)	C13D—C14D—C15D	104.3 (2)
O3B—C9B—C8B	104.74 (19)	C13D—C14D—H14G	110.9
C21B—C9B—C8B	110.2 (2)	C15D—C14D—H14G	110.9
O3B—C9B—C10B	105.0 (2)	C13D—C14D—H14H	110.9
C21B—C9B—C10B	112.3 (2)	C15D—C14D—H14H	110.9
C8B—C9B—C10B	114.6 (2)	H14G—C14D—H14H	108.9
C11B—C10B—C9B	105.0 (2)	C16D—C15D—C14D	103.2 (2)
C11B—C10B—H10D	110.8	C16D—C15D—H15G	111.1
C9B—C10B—H10D	110.8	C14D—C15D—H15G	111.1
C11B—C10B—H10E	110.8	C16D—C15D—H15H	111.1
C9B—C10B—H10E	110.8	C14D—C15D—H15H	111.1
H10D—C10B—H10E	108.8	H15G—C15D—H15H	109.1
C12B—C11B—C10B	102.6 (2)	O5D—C16D—C15D	104.0 (2)
C12B—C11B—H11C	111.2	O5D—C16D—C17D	107.2 (2)
C10B—C11B—H11C	111.2	C15D—C16D—C17D	114.8 (2)
C12B—C11B—H11D	111.2	O5D—C16D—H16D	110.2
C10B—C11B—H11D	111.2	C15D—C16D—H16D	110.2
H11C—C11B—H11D	109.2	C17D—C16D—H16D	110.2
O3B—C12B—O5B	111.6 (2)	O4D—C17D—C19D	111.5 (2)
O3B—C12B—C11B	104.71 (19)	O4D—C17D—C18D	105.1 (2)
O5B—C12B—C11B	104.8 (2)	C19D—C17D—C18D	109.4 (2)
O3B—C12B—C13B	108.4 (2)	O4D—C17D—C16D	107.4 (2)
O5B—C12B—C13B	110.5 (2)	C19D—C17D—C16D	110.9 (2)
C11B—C12B—C13B	116.8 (2)	C18D—C17D—C16D	112.5 (2)
O4B—C13B—C20B	107.3 (2)	C17D—C18D—H18J	109.5
O4B—C13B—C14B	103.10 (19)	C17D—C18D—H18K	109.5
C20B—C13B—C14B	113.6 (2)	H18J—C18D—H18K	109.5
O4B—C13B—C12B	107.6 (2)	C17D—C18D—H18L	109.5
C20B—C13B—C12B	113.8 (2)	H18J—C18D—H18L	109.5
C14B—C13B—C12B	110.6 (2)	H18K—C18D—H18L	109.5
C15B—C14B—C13B	103.9 (2)	C17D—C19D—H19J	109.5
C15B—C14B—H14C	111.0	C17D—C19D—H19K	109.5
C13B—C14B—H14C	111.0	H19J—C19D—H19K	109.5
C15B—C14B—H14D	111.0	C17D—C19D—H19L	109.5
C13B—C14B—H14D	111.0	H19J—C19D—H19L	109.5
H14C—C14B—H14D	109.0	H19K—C19D—H19L	109.5
C14B—C15B—C16B	103.9 (2)	C13D—C20D—H20J	109.5
C14B—C15B—H15C	111.0	C13D—C20D—H20K	109.5
C16B—C15B—H15C	111.0	H20J—C20D—H20K	109.5
C14B—C15B—H15D	111.0	C13D—C20D—H20L	109.5
C16B—C15B—H15D	111.0	H20J—C20D—H20L	109.5
H15C—C15B—H15D	109.0	H20K—C20D—H20L	109.5
O4B—C16B—C17B	107.9 (2)	C9D—C21D—H21J	109.5
O4B—C16B—C15B	103.4 (2)	C9D—C21D—H21K	109.5
C17B—C16B—C15B	114.4 (2)	H21J—C21D—H21K	109.5
O4B—C16B—H16B	110.3	C9D—C21D—H21L	109.5
C17B—C16B—H16B	110.3	H21J—C21D—H21L	109.5
C15B—C16B—H16B	110.3	H21K—C21D—H21L	109.5
O5B—C17B—C19B	111.7 (2)	C4D—C22D—H22J	109.5
O5B—C17B—C18B	104.2 (2)	C4D—C22D—H22K	109.5
C19B—C17B—C18B	109.6 (2)	H22J—C22D—H22K	109.5
O5B—C17B—C16B	107.5 (2)	C4D—C22D—H22L	109.5
C19B—C17B—C16B	110.6 (2)	H22J—C22D—H22L	109.5
C18B—C17B—C16B	113.0 (2)	H22K—C22D—H22L	109.5
C17B—C18B—H18D	109.5	C1A—O1A—C4A	110.8 (2)
C17B—C18B—H18E	109.5	C5A—O2A—C8Y	108.78 (19)
H18D—C18B—H18E	109.5	C5A—O2A—C8A	108.78 (19)
C17B—C18B—H18F	109.5	C12A—O3A—C9A	110.16 (17)
H18D—C18B—H18F	109.5	C16A—O4A—C13A	102.7 (2)
H18E—C18B—H18F	109.5	C12A—O5A—C17A	118.15 (19)
C17B—C19B—H19D	109.5	C1B—O1B—C4B	110.8 (2)
C17B—C19B—H19E	109.5	C5B—O2B—C8B	108.44 (19)
H19D—C19B—H19E	109.5	C12B—O3B—C9B	110.17 (18)
C17B—C19B—H19F	109.5	C16B—O4B—C13B	103.25 (18)
H19D—C19B—H19F	109.5	C12B—O5B—C17B	117.76 (18)
H19E—C19B—H19F	109.5	C1C—O1C—C4C	111.3 (2)
C13B—C20B—H20D	109.5	C5C—O2C—C8C	109.57 (19)
C13B—C20B—H20E	109.5	C12C—O3C—C9C	110.93 (17)
H20D—C20B—H20E	109.5	C16C—O4C—C13C	103.44 (19)
C13B—C20B—H20F	109.5	C12C—O5C—C17C	117.92 (18)
H20D—C20B—H20F	109.5	C1D—O1D—C4D	111.2 (2)
H20E—C20B—H20F	109.5	C5D—O2D—C8D	108.96 (18)
C9B—C21B—H21D	109.5	C12D—O3D—C9D	110.62 (17)
C9B—C21B—H21E	109.5	C12D—O4D—C17D	118.42 (19)
H21D—C21B—H21E	109.5	C13D—O5D—C16D	103.79 (19)
C9B—C21B—H21F	109.5		
			
O6A—C1A—C2A—C3A	−166.6 (3)	O4C—C16C—C17C—C18C	−63.7 (3)
O1A—C1A—C2A—C3A	11.8 (3)	C15C—C16C—C17C—C18C	−178.4 (2)
C1A—C2A—C3A—C4A	−21.2 (3)	O4C—C16C—C17C—C19C	173.5 (2)
C2A—C3A—C4A—O1A	23.0 (3)	C15C—C16C—C17C—C19C	58.9 (3)
C2A—C3A—C4A—C22A	138.7 (3)	O6D—C1D—C2D—C3D	−170.3 (3)
C2A—C3A—C4A—C5A	−94.6 (3)	O1D—C1D—C2D—C3D	8.6 (3)
O1A—C4A—C5A—O2A	−64.4 (3)	C1D—C2D—C3D—C4D	−19.5 (3)
C22A—C4A—C5A—O2A	179.3 (2)	C2D—C3D—C4D—O1D	23.2 (3)
C3A—C4A—C5A—O2A	50.9 (3)	C2D—C3D—C4D—C22D	140.0 (2)
O1A—C4A—C5A—C6Y	55.5 (3)	C2D—C3D—C4D—C5D	−93.2 (3)
C22A—C4A—C5A—C6Y	−60.8 (3)	O1D—C4D—C5D—O2D	−66.3 (2)
C3A—C4A—C5A—C6Y	170.7 (2)	C22D—C4D—C5D—O2D	176.2 (2)
O1A—C4A—C5A—C6A	55.5 (3)	C3D—C4D—C5D—O2D	48.0 (3)
C22A—C4A—C5A—C6A	−60.8 (3)	O1D—C4D—C5D—C6D	50.7 (3)
C3A—C4A—C5A—C6A	170.7 (2)	C22D—C4D—C5D—C6D	−66.7 (3)
O2A—C5A—C6A—C7A	−25.8 (7)	C3D—C4D—C5D—C6D	165.0 (2)
C4A—C5A—C6A—C7A	−146.5 (6)	O2D—C5D—C6D—C7D	−32.8 (3)
C5A—C6A—C7A—C8A	21.2 (10)	C4D—C5D—C6D—C7D	−152.1 (2)
C6A—C7A—C8A—O2A	−9.8 (10)	C5D—C6D—C7D—C8D	20.5 (3)
C6A—C7A—C8A—C9A	128.4 (5)	C6D—C7D—C8D—O2D	−1.2 (3)
O2A—C5A—C6Y—C7Y	4.6 (4)	C6D—C7D—C8D—C9D	118.2 (2)
C4A—C5A—C6Y—C7Y	−116.2 (3)	O2D—C8D—C9D—O3D	−175.31 (17)
C5A—C6Y—C7Y—C8Y	−25.7 (5)	C7D—C8D—C9D—O3D	65.8 (3)
C6Y—C7Y—C8Y—O2A	37.6 (5)	O2D—C8D—C9D—C21D	−57.5 (2)
C6Y—C7Y—C8Y—C9A	153.4 (3)	C7D—C8D—C9D—C21D	−176.4 (2)
C9A—C10A—C11A—C12A	11.0 (16)	O2D—C8D—C9D—C10D	69.8 (3)
C9A—C10Y—C11Y—C12A	29.0 (4)	C7D—C8D—C9D—C10D	−49.1 (3)
O2A—C8A—C9A—O3A	−177.29 (19)	O3D—C9D—C10D—C11D	−6.8 (3)
C7A—C8A—C9A—O3A	44.2 (6)	C21D—C9D—C10D—C11D	−125.7 (2)
O2A—C8A—C9A—C21A	−62.3 (3)	C8D—C9D—C10D—C11D	108.2 (2)
C7A—C8A—C9A—C21A	159.2 (6)	C9D—C10D—C11D—C12D	24.6 (3)
O2A—C8Y—C9A—O3A	−177.29 (19)	C10D—C11D—C12D—O3D	−33.9 (3)
C7Y—C8Y—C9A—O3A	70.8 (3)	C10D—C11D—C12D—O4D	83.2 (2)
O2A—C8Y—C9A—C21A	−62.3 (3)	C10D—C11D—C12D—C13D	−154.4 (2)
C7Y—C8Y—C9A—C21A	−174.2 (3)	O3D—C12D—C13D—O5D	69.0 (2)
C11Y—C10Y—C9A—O3A	−15.5 (4)	O4D—C12D—C13D—O5D	−53.9 (3)
C11Y—C10Y—C9A—C21A	−130.9 (3)	C11D—C12D—C13D—O5D	−173.3 (2)
C11A—C10A—C9A—O3A	11.0 (13)	O3D—C12D—C13D—C20D	−49.8 (3)
C11A—C10A—C9A—C21A	−116.5 (11)	O4D—C12D—C13D—C20D	−172.7 (2)
C10A—C11A—C12A—O3A	−29.1 (13)	C11D—C12D—C13D—C20D	67.9 (3)
C10A—C11A—C12A—O5A	91.6 (12)	O3D—C12D—C13D—C14D	−179.5 (2)
C10A—C11A—C12A—C13A	−143.9 (10)	O4D—C12D—C13D—C14D	57.6 (3)
C10Y—C11Y—C12A—O3A	−32.3 (4)	C11D—C12D—C13D—C14D	−61.8 (3)
C10Y—C11Y—C12A—O5A	82.9 (3)	O5D—C13D—C14D—C15D	27.0 (3)
C10Y—C11Y—C12A—C13A	−155.2 (3)	C20D—C13D—C14D—C15D	143.1 (2)
O3A—C12A—C13A—O4A	65.8 (2)	C12D—C13D—C14D—C15D	−87.6 (3)
O5A—C12A—C13A—O4A	−54.9 (3)	C13D—C14D—C15D—C16D	−0.1 (3)
C11A—C12A—C13A—O4A	176.6 (8)	C14D—C15D—C16D—O5D	−27.1 (3)
C11Y—C12A—C13A—O4A	−173.0 (3)	C14D—C15D—C16D—C17D	89.7 (3)
O3A—C12A—C13A—C20A	−53.3 (3)	O5D—C16D—C17D—O4D	60.5 (3)
O5A—C12A—C13A—C20A	−174.0 (2)	C15D—C16D—C17D—O4D	−54.5 (3)
C11A—C12A—C13A—C20A	57.5 (8)	O5D—C16D—C17D—C19D	−61.5 (3)
C11Y—C12A—C13A—C20A	67.9 (4)	C15D—C16D—C17D—C19D	−176.4 (2)
O3A—C12A—C13A—C14A	178.3 (2)	O5D—C16D—C17D—C18D	175.7 (2)
O5A—C12A—C13A—C14A	57.7 (3)	C15D—C16D—C17D—C18D	60.7 (3)
C11A—C12A—C13A—C14A	−70.8 (8)	O6A—C1A—O1A—C4A	−178.2 (2)
C11Y—C12A—C13A—C14A	−60.4 (4)	C2A—C1A—O1A—C4A	3.2 (3)
O4A—C13A—C14A—C15A	26.9 (3)	C22A—C4A—O1A—C1A	−137.1 (2)
C20A—C13A—C14A—C15A	142.9 (3)	C3A—C4A—O1A—C1A	−16.7 (3)
C12A—C13A—C14A—C15A	−88.8 (3)	C5A—C4A—O1A—C1A	104.4 (2)
C13A—C14A—C15A—C16A	0.8 (4)	C6Y—C5A—O2A—C8Y	20.0 (3)
C14A—C15A—C16A—O4A	−28.4 (3)	C4A—C5A—O2A—C8Y	145.8 (2)
C14A—C15A—C16A—C17A	88.7 (3)	C6A—C5A—O2A—C8A	20.0 (3)
O4A—C16A—C17A—O5A	60.5 (3)	C4A—C5A—O2A—C8A	145.8 (2)
C15A—C16A—C17A—O5A	−54.0 (3)	C9A—C8Y—O2A—C5A	−154.2 (2)
O4A—C16A—C17A—C19A	−61.4 (3)	C7Y—C8Y—O2A—C5A	−36.0 (3)
C15A—C16A—C17A—C19A	−176.0 (3)	C9A—C8A—O2A—C5A	−154.2 (2)
O4A—C16A—C17A—C18A	174.7 (3)	C7A—C8A—O2A—C5A	−6.4 (7)
C15A—C16A—C17A—C18A	60.1 (4)	O5A—C12A—O3A—C9A	−85.7 (2)
O6B—C1B—C2B—C3B	−170.4 (3)	C11A—C12A—O3A—C9A	39.2 (8)
O1B—C1B—C2B—C3B	8.0 (3)	C11Y—C12A—O3A—C9A	23.6 (4)
C1B—C2B—C3B—C4B	−19.1 (3)	C13A—C12A—O3A—C9A	154.0 (2)
C2B—C3B—C4B—O1B	23.0 (3)	C8A—C9A—O3A—C12A	−133.4 (2)
C2B—C3B—C4B—C22B	139.3 (2)	C8Y—C9A—O3A—C12A	−133.4 (2)
C2B—C3B—C4B—C5B	−93.3 (3)	C10Y—C9A—O3A—C12A	−5.1 (4)
O1B—C4B—C5B—O2B	−66.5 (3)	C10A—C9A—O3A—C12A	−31.6 (8)
C22B—C4B—C5B—O2B	176.6 (2)	C21A—C9A—O3A—C12A	110.9 (3)
C3B—C4B—C5B—O2B	47.7 (3)	C17A—C16A—O4A—C13A	−75.3 (3)
O1B—C4B—C5B—C6B	51.4 (3)	C15A—C16A—O4A—C13A	46.6 (3)
C22B—C4B—C5B—C6B	−65.5 (3)	C20A—C13A—O4A—C16A	−165.8 (2)
C3B—C4B—C5B—C6B	165.6 (2)	C14A—C13A—O4A—C16A	−46.0 (3)
O2B—C5B—C6B—C7B	−21.8 (3)	C12A—C13A—O4A—C16A	71.6 (2)
C4B—C5B—C6B—C7B	−141.2 (3)	O3A—C12A—O5A—C17A	−77.1 (3)
C5B—C6B—C7B—C8B	5.4 (4)	C11A—C12A—O5A—C17A	166.4 (6)
C6B—C7B—C8B—O2B	12.8 (3)	C11Y—C12A—O5A—C17A	171.6 (3)
C6B—C7B—C8B—C9B	131.9 (3)	C13A—C12A—O5A—C17A	42.4 (3)
O2B—C8B—C9B—O3B	−173.04 (18)	C19A—C17A—O5A—C12A	77.2 (3)
C7B—C8B—C9B—O3B	68.8 (3)	C16A—C17A—O5A—C12A	−44.9 (3)
O2B—C8B—C9B—C21B	−55.4 (3)	C18A—C17A—O5A—C12A	−164.3 (3)
C7B—C8B—C9B—C21B	−173.6 (2)	O6B—C1B—O1B—C4B	−174.4 (3)
O2B—C8B—C9B—C10B	72.5 (3)	C2B—C1B—O1B—C4B	7.0 (3)
C7B—C8B—C9B—C10B	−45.7 (3)	C22B—C4B—O1B—C1B	−139.8 (2)
O3B—C9B—C10B—C11B	−8.9 (3)	C5B—C4B—O1B—C1B	101.0 (2)
C21B—C9B—C10B—C11B	−127.8 (2)	C3B—C4B—O1B—C1B	−19.0 (3)
C8B—C9B—C10B—C11B	105.5 (3)	C4B—C5B—O2B—C8B	156.4 (2)
C9B—C10B—C11B—C12B	26.6 (3)	C6B—C5B—O2B—C8B	30.9 (3)
C10B—C11B—C12B—O3B	−35.6 (3)	C9B—C8B—O2B—C5B	−153.4 (2)
C10B—C11B—C12B—O5B	82.0 (2)	C7B—C8B—O2B—C5B	−27.5 (3)
C10B—C11B—C12B—C13B	−155.5 (2)	O5B—C12B—O3B—C9B	−81.2 (2)
O3B—C12B—C13B—O4B	67.3 (2)	C11B—C12B—O3B—C9B	31.7 (3)
O5B—C12B—C13B—O4B	−55.2 (2)	C13B—C12B—O3B—C9B	157.00 (19)
C11B—C12B—C13B—O4B	−174.8 (2)	C21B—C9B—O3B—C12B	106.5 (2)
O3B—C12B—C13B—C20B	−51.5 (3)	C8B—C9B—O3B—C12B	−135.3 (2)
O5B—C12B—C13B—C20B	−174.0 (2)	C10B—C9B—O3B—C12B	−14.2 (3)
C11B—C12B—C13B—C20B	66.4 (3)	C17B—C16B—O4B—C13B	−75.4 (2)
O3B—C12B—C13B—C14B	179.24 (19)	C15B—C16B—O4B—C13B	46.2 (2)
O5B—C12B—C13B—C14B	56.7 (3)	C20B—C13B—O4B—C16B	−165.8 (2)
C11B—C12B—C13B—C14B	−62.9 (3)	C14B—C13B—O4B—C16B	−45.6 (2)
O4B—C13B—C14B—C15B	26.8 (3)	C12B—C13B—O4B—C16B	71.3 (2)
C20B—C13B—C14B—C15B	142.6 (2)	O3B—C12B—O5B—C17B	−77.2 (3)
C12B—C13B—C14B—C15B	−88.1 (3)	C11B—C12B—O5B—C17B	170.0 (2)
C13B—C14B—C15B—C16B	0.6 (3)	C13B—C12B—O5B—C17B	43.4 (3)
C14B—C15B—C16B—O4B	−27.9 (3)	C19B—C17B—O5B—C12B	76.3 (3)
C14B—C15B—C16B—C17B	89.2 (3)	C18B—C17B—O5B—C12B	−165.4 (2)
O4B—C16B—C17B—O5B	60.4 (3)	C16B—C17B—O5B—C12B	−45.2 (3)
C15B—C16B—C17B—O5B	−54.0 (3)	O6C—C1C—O1C—C4C	−176.0 (2)
O4B—C16B—C17B—C19B	−61.8 (3)	C2C—C1C—O1C—C4C	5.4 (3)
C15B—C16B—C17B—C19B	−176.3 (2)	C5C—C4C—O1C—C1C	100.6 (2)
O4B—C16B—C17B—C18B	174.8 (2)	C22C—C4C—O1C—C1C	−139.9 (2)
C15B—C16B—C17B—C18B	60.4 (3)	C3C—C4C—O1C—C1C	−19.8 (3)
O6C—C1C—C2C—C3C	−167.1 (3)	C4C—C5C—O2C—C8C	152.4 (2)
O1C—C1C—C2C—C3C	11.4 (3)	C6C—C5C—O2C—C8C	27.5 (3)
C1C—C2C—C3C—C4C	−22.6 (3)	C7C—C8C—O2C—C5C	−8.7 (3)
C2C—C3C—C4C—O1C	25.6 (3)	C9C—C8C—O2C—C5C	−135.8 (2)
C2C—C3C—C4C—C5C	−91.3 (3)	O5C—C12C—O3C—C9C	−86.2 (2)
C2C—C3C—C4C—C22C	142.2 (2)	C11C—C12C—O3C—C9C	27.8 (3)
O1C—C4C—C5C—O2C	−58.7 (3)	C13C—C12C—O3C—C9C	152.8 (2)
C22C—C4C—C5C—O2C	−176.7 (2)	C10C—C9C—O3C—C12C	−15.8 (3)
C3C—C4C—C5C—O2C	55.5 (3)	C8C—C9C—O3C—C12C	−138.7 (2)
O1C—C4C—C5C—C6C	59.1 (3)	C21C—C9C—O3C—C12C	105.6 (2)
C22C—C4C—C5C—C6C	−58.8 (3)	C15C—C16C—O4C—C13C	47.5 (2)
C3C—C4C—C5C—C6C	173.4 (2)	C17C—C16C—O4C—C13C	−74.5 (2)
O2C—C5C—C6C—C7C	−34.9 (3)	C20C—C13C—O4C—C16C	−165.2 (2)
C4C—C5C—C6C—C7C	−155.4 (2)	C12C—C13C—O4C—C16C	72.8 (2)
C5C—C6C—C7C—C8C	29.5 (3)	C14C—C13C—O4C—C16C	−44.2 (2)
C6C—C7C—C8C—O2C	−13.7 (3)	O3C—C12C—O5C—C17C	−76.2 (3)
C6C—C7C—C8C—C9C	108.3 (3)	C11C—C12C—O5C—C17C	170.8 (2)
O2C—C8C—C9C—O3C	−177.62 (19)	C13C—C12C—O5C—C17C	44.4 (3)
C7C—C8C—C9C—O3C	61.7 (3)	C18C—C17C—O5C—C12C	77.1 (3)
O2C—C8C—C9C—C10C	66.8 (3)	C19C—C17C—O5C—C12C	−164.9 (2)
C7C—C8C—C9C—C10C	−53.9 (3)	C16C—C17C—O5C—C12C	−44.8 (3)
O2C—C8C—C9C—C21C	−61.6 (3)	O6D—C1D—O1D—C4D	−174.2 (2)
C7C—C8C—C9C—C21C	177.7 (2)	C2D—C1D—O1D—C4D	6.7 (3)
O3C—C9C—C10C—C11C	−3.1 (3)	C22D—C4D—O1D—C1D	−139.5 (2)
C8C—C9C—C10C—C11C	112.4 (3)	C5D—C4D—O1D—C1D	101.3 (2)
C21C—C9C—C10C—C11C	−121.6 (3)	C3D—C4D—O1D—C1D	−19.0 (3)
C9C—C10C—C11C—C12C	19.0 (3)	C6D—C5D—O2D—C8D	33.5 (2)
C10C—C11C—C12C—O3C	−28.1 (3)	C4D—C5D—O2D—C8D	158.65 (19)
C10C—C11C—C12C—O5C	89.5 (3)	C9D—C8D—O2D—C5D	−146.59 (19)
C10C—C11C—C12C—C13C	−148.2 (2)	C7D—C8D—O2D—C5D	−20.3 (2)
O3C—C12C—C13C—O4C	64.9 (3)	O4D—C12D—O3D—C9D	−81.1 (2)
O5C—C12C—C13C—O4C	−57.1 (3)	C11D—C12D—O3D—C9D	31.3 (2)
C11C—C12C—C13C—O4C	−177.4 (2)	C13D—C12D—O3D—C9D	156.59 (19)
O3C—C12C—C13C—C20C	−53.5 (3)	C21D—C9D—O3D—C12D	105.7 (2)
O5C—C12C—C13C—C20C	−175.5 (2)	C8D—C9D—O3D—C12D	−135.87 (19)
C11C—C12C—C13C—C20C	64.2 (3)	C10D—C9D—O3D—C12D	−15.4 (3)
O3C—C12C—C13C—C14C	176.7 (2)	O3D—C12D—O4D—C17D	−79.3 (3)
O5C—C12C—C13C—C14C	54.7 (3)	C11D—C12D—O4D—C17D	168.7 (2)
C11C—C12C—C13C—C14C	−65.6 (3)	C13D—C12D—O4D—C17D	42.4 (3)
O4C—C13C—C14C—C15C	23.8 (3)	C19D—C17D—O4D—C12D	76.5 (3)
C20C—C13C—C14C—C15C	140.1 (2)	C18D—C17D—O4D—C12D	−165.0 (2)
C12C—C13C—C14C—C15C	−90.6 (3)	C16D—C17D—O4D—C12D	−45.1 (3)
C13C—C14C—C15C—C16C	4.1 (3)	C20D—C13D—O5D—C16D	−166.1 (2)
C14C—C15C—C16C—O4C	−30.7 (3)	C14D—C13D—O5D—C16D	−45.4 (2)
C14C—C15C—C16C—C17C	86.4 (3)	C12D—C13D—O5D—C16D	71.4 (2)
O4C—C16C—C17C—O5C	58.6 (3)	C15D—C16D—O5D—C13D	46.0 (2)
C15C—C16C—C17C—O5C	−56.0 (3)	C17D—C16D—O5D—C13D	−76.0 (2)

### Hydrogen-bond geometry (Å, º)

**Table:**

*D*—H···*A*	*D*—H	H···*A*	*D*···*A*	*D*—H···*A*
C3*C*—H3*C*1···O6*B*^i^	0.99	2.64	3.300 (4)	125
C22*D*—H22*K*···O6*A*^ii^	0.98	2.55	3.400 (3)	146
C2*B*—H2*B*2···O6*D*^iii^	0.99	2.62	3.423 (4)	139
C22*B*—H22*E*···O6*C*^iv^	0.98	2.68	3.501 (4)	142
C5*B*—H5*B*···O4*A*	1.00	2.66	3.510 (3)	143
C2*D*—H2*D*2···O6*C*^v^	0.99	2.67	3.573 (4)	152
C16*D*—H16*D*···O1*B*^i^	1.00	2.61	3.591 (3)	166

Symmetry codes: (i) −*x*+1, −*y*+1, −*z*; (ii) −*x*, −*y*, −*z*+1; (iii) *x*, *y*, *z*−1; (iv) −*x*, −*y*+1, −*z*; (v) −*x*, −*y*+1, −*z*+1.
